# Intervention in Neuropsychiatric Disorders by Suppressing Inflammatory and Oxidative Stress Signal and Exploration of In Silico Studies for Potential Lead Compounds from *Holigarna caustica* (Dennst.) Oken leaves

**DOI:** 10.3390/biom10040561

**Published:** 2020-04-06

**Authors:** Md. Adnan, Md. Nazim Uddin Chy, A.T.M. Mostafa Kamal, Kazi Asfak Ahmed Chowdhury, Md. Atiar Rahman, A. S. M. Ali Reza, Md. Moniruzzaman, Satyajit Roy Rony, Mst. Samima Nasrin, Md Obyedul Kalam Azad, Cheol Ho Park, Young Seok Lim, Dong Ha Cho

**Affiliations:** 1 Department of Bio-Health Technology, Kangwon National University, Chuncheon 24341, Korea; mdadnan1991.pharma@gmail.com (M.A.); azadokalam@gmail.com (M.O.K.A.); chpark@kangwon.ac.kr (C.H.P.); 2 Department of Pharmacy, International Islamic University Chittagong, Chittagong 4318, Bangladesh; nazim107282@gmail.com (M.N.U.C.); ashfak4u_ctg@yahoo.com (K.A.A.C.); alirezaru@gmail.com (A.S.M.A.R.); shathy_ru@yahoo.com (M.S.N.); 3 Drug Discovery, GUSTO A Research Group, Chittagong 4000, Bangladesh; 4 Department of Biochemistry & Molecular Biology, University of Chittagong, Chittagong 4331, Bangladesh; atiar@cu.ac.bd; 5 Designated Reference Institute for Chemical Measurement (DRiCM), Bangladesh Council of Scientific & Industrial Research (BCSIR), Dhaka 1205, Bangladesh; monir.accedu@gmail.com; 6 BCSIR Laboratories, Chittagong, Bangladesh Council of Scientific & Industrial Research (BCSIR), Chittagong 4220, Bangladesh; satyajit_pharm@bcsir.gov.bd

**Keywords:** *Holigarna caustica* (Dennst.), anxiolytic, antidepressant, anti-inflammatory, antioxidant, molecular docking, ADME/T, admetSAR and PASS

## Abstract

*Holigarna caustica* (Dennst.), a popular plant used in folk medicine in Bangladesh, is often used by the local folk practitioner to treat a variety of chronic diseases. The present research is an attempt to find out an innovative therapeutic prospect for the management of neuropsychiatric disorders. The methanol extract of *H. caustica* leaves (MEHC) were utilized on various behavioral tests for assessing anxiolytic, anti-depressant, and anti-inflammatory activities. The antioxidant potentials and quantitative phytochemicals were evaluated through spectrophotometric methods. Results revealed that treatment of MEHC (200 and 400 mg/kg) significantly reduced anxiety like behaviors in mice, particularly, 400 mg/kg efficiently improved % of entries and time spent (*p* < 0.05) in the open arms in elevated plus maze test, whereas, superior head dipping tendency (*p* < 0.05) was observed in hole-board test. In contrast, mice treated with 200 mg/kg revealed better anxiolytic effect in both open field and hole-cross tests. During antidepressant evaluation, mice administrated with MEHC exhibited active behaviors (swimming and struggling) in forced swimming and tail suspension tests. In parallel, MEHC manifested a noteworthy (*p* < 0.001) suppression of inflammatory response induced by histamine. The MEHC also showed strong antioxidant activities in 1,1-diphenyl-2-picrylhydrazyl radical (DPPH) (IC_50_: 57.64 μg/mL) scavenging, H_2_O_2_ (IC_50_: 51.60 μg/mL) scavenging, and ferric reducing power assay. The levels of total phenol, flavonoid, flavonol, condensed tannin, and antioxidant were estimated as higher in MEHC. Moreover, 11 compounds were documented as bioactive, displayed good binding affinities to potassium channel receptor, human serotonin receptor, cyclooxygenase (COX-1 and 2), and xanthine oxidoreductase enzyme targets in molecular docking experiments. Furthermore, ADME/T and Prediction of Activity Spectra for Substances (PASS) analyses exposed their drug-likeness, nontoxic upon consumption, and likely pharmacological actions. Overall, the H. caustica is potentially bioactive as evident by in vivo, in vitro, and computational analysis. Our findings support the folkloric value of this plant, which may provide a potential source towards developing drug leads.

## 1. Introduction

In recent years, mental and behavioral disarrays are among the vital principle of disability due to the interference of affected people’s mood and emotion [1]. According to the Study highlight (2017) of The Global Burden of Disease, Injuries, and Risk Factors (GBD), neuropsychiatric disorders are ranked as the third leading cause of disability, where the suffering rate are higher in the case of females [2]. Besides, The World Health Organization (WHO) categorized depressive disorders as the major contributor to non-fatal health suffering worldwide, and anxiety disorders possess the sixth position [3]. 

Depression is a persistent and multiplex disorder with an expansive impact on the community and is connected with functional disablement and elevated morbidity and mortality; in contrast, anxiety is another frequent psychiatric disorder globally [4]. It is suggested by different corroboration that depressive and anxiety disorders coincide and do not constitute discrete disease entities. Indeed, approximately one-half of those investigated with depression are additionally diagnosed with anxiety disarray. The existence of anxiety in accord with depression leads into complications of symptoms, less authentic prognosis, worsened response for treatment or treatment dropout, and a higher risk of suicidal tendency [5]. 

The actual etiology of anxiety and depression remains a great enigma, but some dominant factors such as genetic, environmental, biological, and psychological have been unfolded to be involved in the progression of such neuropsychiatric disorders [6]. One of the most potential stimulators of these disorders is chronic pain and inflammation, which have an intense mutual relationship with anxiety and depression. Interestingly, the clinical manifestations, neurotransmitters, pro-inflammatory cytokines, and neurological pathways of nociception and depression have parallel communication [7]. In addition, the mechanisms of neurotransmitters such as serotonin and norepinephrine have a similar role for modulating depression and pain signaling in the brain and nervous system [8]. Hence, patients with chronic pain and inflammation may suffer from anxiety accompanied by a progressive depressive state. Another intimidating issue is the deterioration of antioxidant defense system which induces oxidative stress or redox imbalance, subsequently leading to the neuropsychiatric disorders [9]. The over-production of reactive oxygen species (ROS) in the brain establishes a state of cellular disparity that causes cognitive dysfunctions and impairment of neurobiological mechanisms [10]. Emerging evidence suggests that oxidative stress caused by chronic inflammatory signals not only promotes major depressive disorders (MDD), but also contribute to pro-inflammatory molecules production [11]. Therefore, antioxidant therapies may be required to improve the progressive tissue damage, following the counterbalance of ROS production in the central nervous system. Hence, long-term pharmacotherapy and polypharmacy, such as co-administration of antipsychotic agents with antioxidant supplements, are recommended to treat such critical health issues, which, in this regard, create disinclination of taking medication among the patients.

However, current top-line antidepressant drugs (e.g., benzodiazepines, selective serotonin and/or serotonin-norepinephrine reuptake inhibitors) cannot deliver enough therapeutic interventions to regulate anxiety, depression, chronic inflammation, and ROS simultaneously [12]. Additionally, the great concern is their association of unwanted side effects, including sedation, sexual dysfunction, memory disturbances, abuse liability, amnesia, and daytime drowsiness [13]. In such cases, exploration of potential bioactive compounds from medicinal plants having multifaceted pharmacological targets is the ultimate focus of the current global research interest. Certainly, drug discovery programs from medicinal plants is a challenging and time consuming process [14]. Since drug developments are involved in the discovery of lead compounds from medicinal plants, followed by lead identification (plant procurement, extraction, target based bioassay: in vitro and in vivo), lead optimization (including medicinal and combinatorial chemistry), lead development (involving pharmacology, toxicology, pharmacokinetics, ADME, and drug delivery), and finally, after successful consecutive clinical trials, the compounds are approved for the clinical application [15]. Despite these challenges, medicinal plants derived natural products have been used around the globe clinically, even for the management of the common cold to life threatening conditions [16]. Hence, in our study, we have designed a biological investigation on *Holigarna caustica*, in order to evaluate the bioactive constituents and their multifarious pharmacological potentials to treat the chronic diseases. 

*Holigarna caustica* (Dennst.) Oken (black varnish tree) is a potential medicinal plant, commonly known as “Borola” “katebale” in the regions of Chittagong hill tract, Bangladesh. Traditionally it has a wide range of uses, for instance, management of various painful conditions (e.g., eye irritation, inflammation, and arthritis) with *H. Caustica* is commonplace among different ethnic communities (Khumi and Marma communities). Moreover, the indigenous communities also use *H. Caustica* in mitigating various chronic diseases, such as haemorrhoids and obesity (Tripura), tumors and cancers (Khumi), skin disease and antiseptic (Marma) [17]. To verify their established ethno-medicinal use, we previously investigated the anti-nociceptive and anti-inflammatory potentials as well as their possible mechanism of actions. Besides, GC-MS analysis of methanol extract of *H. Caustica* (MEHC) revealed a total of 40 compounds, among them 12 compounds were documented as bioactive which were reported to possess anti-nociceptive an anti-inflammatory activities [18]. However, our current study has been designed with the aim of systematic explore to further investigate the anxiolytic, antidepressant, anti-inflammatory, and antioxidant activities of this plant. Most importantly, to find the potential therapeutic intervention of MEHC in mitigating neuropsychiatric disorders either by blocking inflammation and/or oxidative stress signal is the ultimate goal of our research. In addition, to integrate the pharmacological responses of MEHC, we have also performed computational (molecular docking, ADME/T, and PASS) studies to unveil the potential target insights of the documented bioactive constituents for the possible prospective lead compounds from MEHC for the very first time. 

## 2. Materials and Methods 

### 2.1. Drugs, Chemicals, and Equipment

Methanol, ferric chloride, aluminum chloride, potassium ferricyanide, sodium carbonate, sodium phosphate, potassium acetate, ammonium molybdate, phosphate buffer, hydrogen peroxide, hydrochloric acid, and sulfuric acid were obtained from Merck (Darmstadt, Germany). Ascorbic acid, histamine, catechin, and quercetin were procured from BDH Chemicals Ltd. (Poole, UK). Vanillin, gallic acid, 1,1-diphenyl-2-picrylhydrazyl radical (DPPH), trichloro-acetic acid (TCA), and Folin-Ciocalteau reagent (FCR) were purchased from Sigma Chemicals Co. (St. Louis, MO, USA). Absorbance was taken using UV-Vis spectrophotometer (UVmini-1240, Shimadzu, Japan). Diazepam, imipramine hydrochloride, and diclofenac sodium were obtained from Square Pharmaceuticals Ltd. (Dhaka, Bangladesh). All other chemicals used in this research were of analytical reagent grade until unless specified with additional reference. 

### 2.2. Plant Collection, Identification, and Preparation of Methanol Extract (MEHC)

The leaves (*Holigarna custica*) were collected, with permission, in the month of October 2016, from Kaptai National Park (22°30′08″N 92°12′04″E), Rangamati district, Chittagong division, Bangladesh. The plant was authenticated by Dr. Shaikh Bokthear Uddin, Professor, Department of Botany, and University of Chittagong, Bangladesh. The voucher specimen (accession no: SUB 1622) was deposited in the Herbarium center of University of Chittagong. The collected leaves were washed, cut, and shade dried (55–60 °C) for a week. The dried leaves were pulverized into a coarse powder through an automated grinder. The fine powder (370 g) was soaked in sufficient methanol (800 mL) for 15 days and vigorously shaken. Then the solution was filtered by using the rotary evaporator and the obtained sticky semi-solid was reserved in 4 °C. 

### 2.3. In Vivo Neuropharmacological Activity

#### 2.3.1. Experimental Animals and Ethical Statements

Adult Swiss Albino mice weighing 25–30 g of both male and female were obtained from Jahangir Nagar University, Savar, Bangladesh. The mice were housed in 120 × 30 × 30 cm polypropylene cages maintained under laboratory conditions (room temperature 25 ± 2 °C; relative humidity 55–60%; 12 h light dark circle; pellets; and clear water). Prior to starting the experiments, the mice were acclimatized (14 days) to adapt with the laboratory environment. The experimental mice were managed according to the “Guide for the Care and use of Laboratory Animals, 8^th^ ed” USA [19]. All experiments were performed in a remote and noiseless ambiance between 9.00 a.m. and 5.00 p.m. All experimental protocols (Pharm-P&D-61/08’16-123) were approved by the institutional animal ethics committee of Department of Pharmacy, International Islamic University Chittagong, Bangladesh. 

#### 2.3.2. Acute Oral Toxicity Test

The acute oral toxicity test was performed using standard laboratory conditions according to the “Organization for Environmental Control Development” guidelines (OECD: Guidelines 420; Fixed Dose Method). The allocated animals (*n* = 6) of each group (control and test) were administered a single oral dose (5, 50, 300, or 2000 mg/kg, body weight) of the test extract (MEHC). Before administration of the extract, mice were kept fasting overnight, and food was also delayed for between 3 and 4 h after administration. All experimental animals were observed individually, with particular monitoring for possible unusual responses including behavioral changes, allergic syndromes (itching, swelling, skin rash), and mortality over the next 72 h.

#### 2.3.3. Experimental Design (Drugs and Treatments)

A total of 24 experimental animals (12 male and 12 female mice for each experiment) were separated into four groups (control, standard, and test groups) containing six mice in each section. The test groups were administrated MEHC at doses of 200 and 400 mg/kg, b.w, p.o, respectively, whereas the control group received vehicle (1% Tween 80 in water, 10 mL/kg, p.o). The standard drug diazepam (1 mg/kg, b.w, i.p) was used in elevated plus maze test, hole-board test, open field test, and hole cross test, while imipramine hydrochloride (10 mg/kg, b.w, i.p) was used for tail suspension test (TST) and forced swim test (FST). The diclofenac sodium (10 mg/kg b.w, p.o) was administrated to the mice of histamine induced paw edema test. Importantly, the reference drugs (diazepam, imipramine hydrochloride, and diclofenac sodium) were administrated at 15 min and MEHC (200 and 400 mg/kg) or vehicle at 30 min prior to the experiments.

#### 2.3.4. Anxiolytic Activity

##### Elevated Plus Maze Test in Mice (EPM)

The elevated plus maze (EPM) test was performed to investigate the anxiolytic activity of MEHC in mice [20]. The apparatus (situated above 40 cm of the floor) used in this test contained two open arms (5 × 10 cm) and two closed arms (5 × 10 × l5 cm) which together merged in a center platform (5 × 5 cm) and exposed as symbol of plus sign. The randomly distributed animals (*n* = 6) of each group were administrated as mentioned in Section 2.3.3. After thirty minutes, each treated animal was kept in the midpoint of the platform, facing to the closed arms and allowed for free roaming for 5 min. During exploration of mice, open arms entrance and total time spent were recorded.

$$\%\;\mathrm{of}\;\mathrm{entries}\;\mathrm{in}\;\mathrm{the}\;\mathrm{open}\;\mathrm{arm}=\frac{\mathrm{No}.\;\mathrm{of}\;\mathrm{entries}\;\mathrm{in}\;\mathrm{the}\;\mathrm{open}\;\mathrm{arm}}{\mathrm{No}.\;\mathrm{of}\;\mathrm{entries}\;\mathrm{in}\;\mathrm{the}\;\mathrm{open}\;\mathrm{arm}+\mathrm{No}.\;\mathrm{of}\;\mathrm{entries}\;\mathrm{in}\;\mathrm{the}\;\mathrm{closed}\;\mathrm{arm}}\times 100$$

##### Hole-Board Test for Exploratory Behavior in Mice (HBT)

In this test, a grid-pattern having sixteen holes (diameter 3 cm) contained a flat platform with an enclosed space (40 × 40 × 25 cm) which was used as an experimental apparatus which was set up 25 cm above the floor. Dosing treatments for each group of animals were described in the section of 2.3.3. Thirty min after post administration of test dose, the experimental animal was situated on the middle of the board and allowed to have free movement. Finally, head dipping number through the holes and latency of head dipping by mice were counted for 5 min [21]. 

#### 2.3.5. Locomotor and Exploration Activity

##### Open Field Test (OFT)

The emotional behavior (locomotor activity) of animals was assessed through the open field test. The instrument and the method applied in this test were obtained as instructed by Gupta et al. (1971) [22]. A wood square box (50 cm × 50 cm × 40 cm) with the floor of half square meter (10 cm × 10 cm) alternatively painted in black and white, which divided into 25 equal squares by lines, was used as experimental apparatus. The categorized animals (*n* = 6) in each group were treated as mentioned in 2.3.3 section. All groups of animals were placed in the middle of the open field, and numbers of squares crossed with all paws (crossing) were counted in a 5 min session at 0, 30, 60, 90, and 120 min intervals.

##### Hole-Cross Test (HCT)

For this investigation, a cage (30 × 20 × 14 cm) was used as an apparatus, having a steel standing partition (containing a 3 cm hole in the center at the height of 7.5 cm) in the middle which divided the cage into two chambers. The allocated animals (*n* = 6) for each group were treated as described in Section 2.3.3. Finally, each animal was placed into the cage and number of passing through the hole from one to another chamber within the cage was recorded for 5 min on 0, 30, 60, 90, and 120 min intervals [23].

#### 2.3.6. Antidepressant Activity

##### Forced Swim Test (FST)

The forced swimming test was carried out to evaluate the antidepressant activity of MEHC in mice, as a previously described method [24]. This experiment was performed in two sessions, for instance, preliminary test was conducted the day before the final experiment in order to adapt the animals with environment. A transparent glass tank (25 × 15 × 25 cm) filled up to 15 cm with water (25 ± 1 °C) was used as an experimental apparatus for swimming. Mice of all groups were treated (tests dose, standard drug, and vehicle) as it was mentioned in 2.3.3 section. After thirty munities, each mouse was placed in the tank for 6 min where first 2 min were considered as initial adjustment time and the next 4 min were recorded as the immobility duration.

##### Tail Suspension Test (TST)

Tail suspension test is the most simple and reliable method to screen the antidepressant activity of MEHC [25]. After administration of all dose as described in Section 2.3.3, mice were induced in a state of depression (immobility), hanging by the end of their tail using adhesive tape (nearly 1 cm from the tip of the tail). The total time of immobility was recorded during the last 4 min of a total 6 min for each mouse of all groups.

### 2.4. Anti-Inflammatory Activity of MEHC in Histamine-Induced Paw Edema Test in Mice

The anti-inflammatory activity of MEHC was assessed following injection of histamine into the plantar surface of the mouse hind paw [26]. Thirty min after the treatment mentioned in Section 2.3.3, histamine (1 mg/kg, in 1% Tween-80 with D.W) was injected (0.05 mL) in the sub-plantar area of the right paw of each mouse to induce acute inflammation, and micrometer slide calipers were used to measure the paw volume at 1–4 h. The inflammatory effect (% inhibition) of the MEHC was determined using the given expression:

$$\%\;\mathrm{inhibition}\;\mathrm{of}\;\mathrm{inflammation}=\frac{\mathrm{Degree}\;\mathrm{of}\;\mathrm{inflammation}\;\left(\mathrm{control}-\mathrm{test}\;\mathrm{group}\right)}{\mathrm{Degree}\;\mathrm{of}\;\mathrm{inflammation}\;\mathrm{of}\;\mathrm{control}}\times 100$$

### 2.5. In Vitro Antioxidant Activity

#### 2.5.1. 1,1-Diphenyl-2-picrylhydrazyl Radical (DPPH) Radical Scavenging Activity 

Free radical scavenging activity of MEHC was assessed using DPPH (1,1 Diphenyl-1-picrylhydrazyl) free radical, following the method of Braca et al [27]. The brief description was explained in Adnan et al., 2018 [28]. The test was conducted in triplicate and results were reported as mean ± SD. 

#### 2.5.2. H_2_O_2_ Scavenging Activity

The hydrogen peroxide scavenging ability of MEHC was determined according to the method of Ruch et al [29]. The brief description was explained in Adnan et al., 2018 [28]. The test was conducted in triplicate and results were reported as mean ± SD.

#### 2.5.3. Ferric Reducing Power Assay (FRPA)

The reducing power of the MEHC was evaluated according to the previously described method [30]. The detailed description was explained in Adnan et al., 2018 [28]. The experiment was analyzed in triplicate and results were reported as mean ± standard error mean (SEM).

### 2.6. Quantitative Phytochemical Analysis

#### 2.6.1. Determination of Total Antioxidant Capacity (TAC)

Total antioxidant activity of the MEHC was estimated by the phosphomolybdate method [31]. The brief description was explained in Adnan et al., 2018 [28]. The experiment was conducted in triplicate and results were reported as mean ± SEM and values are expressed as equivalent of ascorbic acid in mg per g of extract. 

#### 2.6.2. Determination of Total Phenolic Content (TPC)

Total content of phenol in MEHC was determined following the method described previously [32]. The brief explanation was clarified in Adnan et al., 2018 [28]. The TPC was measured from a calibration curve (plotting the value of absorbance vs. concentration) using gallic acid and the results were expressed as mg of GAE (gallic acid equivalent) of the extract. The test was analyzed in triplicate and results were reported as mean ± SEM.

#### 2.6.3. Determination of Total Flavonoid Content

The total flavonoid content of MEHC was evaluated as described by Olayinka A Aiyegoro and Anthony I Okoh [33]. The detailed method was mentioned in Adnan et al., 2018 [28]. The experiment was analyzed in triplicate and results were reported as mean ± SEM and expressed in mg QE/g of the extract.

#### 2.6.4. Determination of Total Flavonol Content 

Total flavonol content of MEHC was determined by adopting the procedure described by Kumaran and Karunakaran [34]. The test was conducted in triplicate and results were reported as mean ± SEM and total flavonol content was calculated as mg/g of quercetin equivalent from the calibration curve. 

#### 2.6.5. Determination of Total Proanthocyanidin Content 

Total proanthocyanidin of MEHC was determined based on the procedure of Oyedemi [35]. The experiment was conducted in triplicate and results were reported as mean ± SEM. Total proanthocyanidin content was evaluated at a concentration of 1 mg/ml and expressed as catechin equivalent (mg/g) using the calibration curve. 

### 2.7. Chemical Compounds Studied in this Article

*β*-D-Glucopyranoside, methyl (PubChem CID:445238), Neophytadiene (PubChem CID:10446), 2-Pentadecanone, 6,10,14-trimethyl (PubChem CID:10408), Hexadecanoic acid, methyl ester (PubChem CID:8181), *n*-Hexadecanoic acid (PubChem CID:985), *α*-Tocospiro A (PubChem CID:21674156), *β*-Sitosterol acetate (PubChem CID:5354503), Vitamin E (PubChem CID:14985), Campesterol (PubChem CID:173183), Stigmasterol (PubChem CID:5280794), and Elaidic acid (PubChem CID: 637517).

### 2.8. In Silico Studies

#### 2.8.1. Molecular Docking Analysis: Ligand Preparation

The chemical structures of eleven major compounds of MEHC were downloaded from PubChem compound repository (https://pubchem.ncbi.nlm.nih.gov/). The ligand was prepared by using the LigPrep tool, which was embedded in Schrödinger suite-Maestro v 10.1, where the following parameters were used as follows: neutralized at pH 7.0 ± 2.0 using Epik 2.2 and the OPLS_2005 force field were used for minimization. 

#### 2.8.2. Molecular Docking Analysis: Enzyme/Receptor Preparation 

Three-dimensional crystallographic structures of enzyme/receptors were obtained from the Protein Data Bank RCSB PDB [36]: potassium channel receptor (PDB: 4UUJ) [37], human serotonin receptor (PDB: 5I6X) [38], cyclooxygenase-1 (COX-1, PDB: 2OYE) [39], cyclooxygenase-2 (COX-2, PDB: 3HS5) [40], and xanthine oxidoreductase enzyme (PDB: 1R4U) [41]. The enzyme/receptor was prepared for a docking experiment using Protein Preparation Wizard [42], which embedded in Schrödinger suite-Maestro v 10.1, as we described previously [43]. 

#### 2.8.3. Molecular Docking Analysis: Glide Standard Precision Docking 

Molecular docking study was made to reveal the possible mechanism of action of the selected compounds behind the biological activities of the MEHC against the respective enzymes/receptor for an anxiolytic, antidepressant, anti-inflammatory, and antioxidant activity. Docking experiments were performed using Glide standard precision docking, which was embedded in Schrödinger suite-Maestro v 10.1, as we described previously [26]. 

#### 2.8.4. In Silico Study: Determination of Pharmacokinetic Parameters by SwissADME

The pharmacokinetic parameters or drug-likeness properties of the selected compounds were determined by SwissADME online tool (http://www.swissadme.ch/). In the present study, an orally active drug should fulfill the following drug-likeness parameters to demonstrate their pharmaceutical fidelity such as molecular weight of the compounds, lipophilicity (LogP), the number of hydrogen-bond acceptors, the number of hydrogen-bond donors, topological polar surface area (TPSA), and the number of rotatable bonds (nRB) based on the Lipinski’s and Veber’s rules. 

#### 2.8.5. In Silico Study: Toxicological Properties Prediction by AdmetSAR 

Toxicological properties of the selected compounds were determined using the admetSAR online tool [44] sine toxicity is a prime concern during the development of new drugs. In the present study, ames toxicity, carcinogenic properties, acute oral toxicity, and rat acute toxicity were predicted. 

#### 2.8.6. In Silico Study: Prediction of Activity Spectra for Substances (PASS) Study by PASS Online 

The six major selected phytocompounds viz. *β*-D Glucopyranoside, methyl, *α*-Tocospiro A, *β*-Sitosterol acetate, campesterol, stigmasterol, and vitamin E were examined for evaluating the anxiolytic, antidepressant, anti-inflammatory, antioxidant, and other biological activities by using PASS online (http://www.pharmaexpert.ru/passonline/).

### 2.9. Statistical Analysis

The data were expressed as mean ± standard error of mean (SEM) and standard deviation (SD) where the p-value less than 0.05, 0.01, and 0.001 were considered as statistically significant. SPSS version 20 software was used for data analysis and all comparisons were performed using one-way ANOVA followed by Dunnett’s multiple comparison tests. 

## 3. Results and Discussion

The primary health care system of the developing countries is apparently contingent with the herbal therapies [45]. Researchers believe that herbal medicines are the opulent source of biomolecules, having multifaceted pharmacological targets, and providing a counterpoise novel mechanistic pathway for the treatment of various chronic diseases [46]. In recent times, a number of potent herbal medicines have been documented and approved by the regulatory bodies for the treatment of anxiety and depression disorders [47]. Several well recognized medicinal plants having anxiolytic and/or antidepressant properties, including kava (*Piper methysticum*) [48], brahmi (*Bacopa monnieri*) [49], passionflower (*Passiflora incarnata*) [50], black cohosh (*Cimicifuga racemosa*) [51], and saffron (*Crocus sativus*) [52], are compared to the standard pharmaceutical agents [53]. The inherent anti-psychotic potentials of these medicinal plants have been evaluated through the vigilant choice of a medicinal plant (careful ethnopharmacological survey), followed by the phytochemical and pharmacological explorations [54]. 

To develop a top-class herbal anxiolytic and/or antidepressant drug, a pragmatic approach with various animal behavior tools is inevitable. Even though an impressive number of animal models are available for the neurobiological research, each experimental model has its pros and cons [55]. Therefore, syndrome based approaches, use of innovative animal prototypes, and well validated tests either successively relevant or in equivalent to pathological psychiatric complaints, may contribute to get a reliable preclinical and clinical conclusions [56]. These elucidations will ultimately facilitate developing classical anxiolytic and/or antidepressant lead compounds from medicinal plants. Cognizant of theses principle, we have adopted such rational approaches to evaluate anxiolytic and antidepressant potentials from the methanol extract of *Holigarna caustica*, utilizing suitable animal behavior models which accurately reflect various aspects of human psychopathology. Before starting the in vivo experiments, the acute toxicity test was conducted in order to evaluate the toxic profile of MEHC. During acute toxicity assay, all measured doses (5 to 2000 mg/kg) did not expose any noticeable indication of toxicity, behavioral abnormalities, and potential defects on motor activities (excitability and sedation). Moreover, overt toxicological effects, particularly, physical changes (allergic reaction and loss of body weight) were not observed, which confirmed that MEHC has no toxic effects up to 2000 mg/kg. 

### 3.1. Anxiolytic Activity

**Figure 1:**
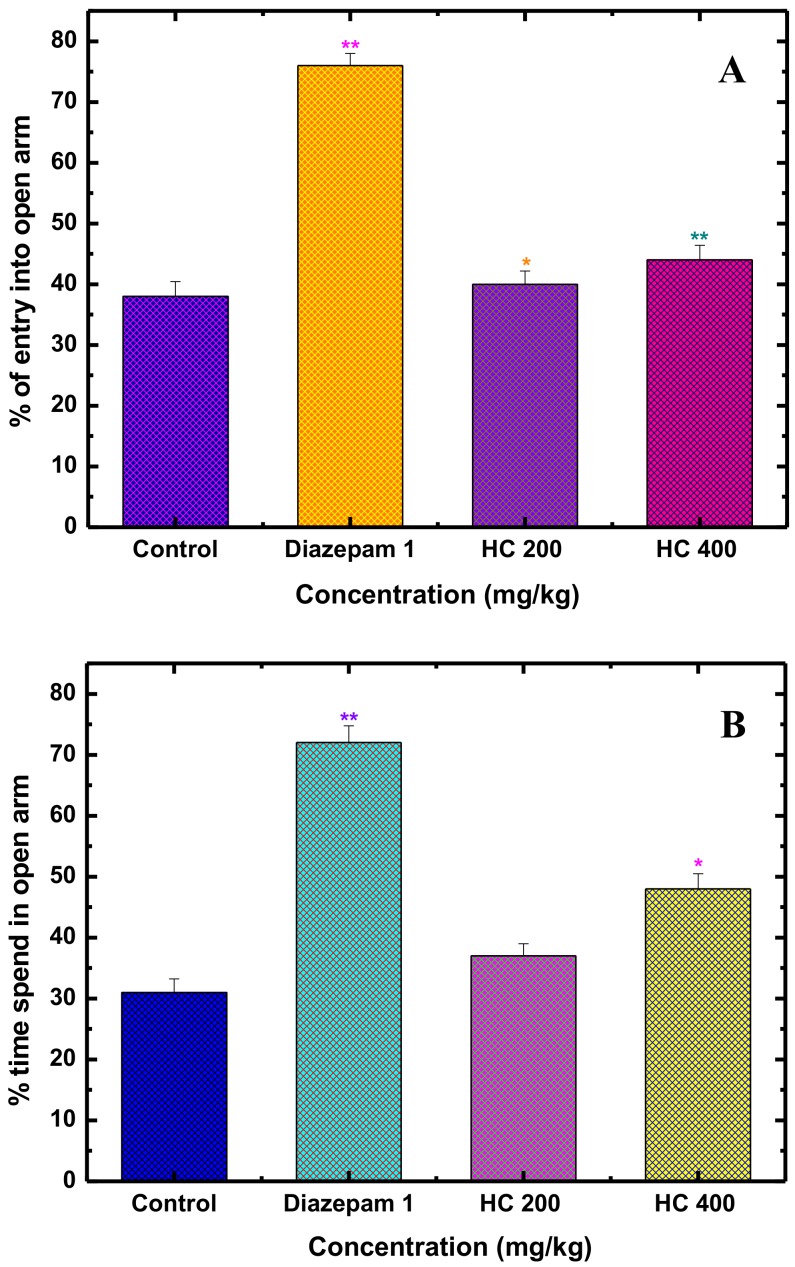
Anxiolytic activity of methanol extract of *H. caustica* (MEHC) and diazepam on elevated plus maze test in mice. % of entry into open arm (**A**), % time spends in open arm (**B**). Values are mean ± S.E.M. (* *p* < 0.05) and (** *p* < 0.01), significantly different from control; ANOVA followed by Dunnett’s test (*n* = 6, per group). MEHC: methanol extract of *Holigarna caustica*; HC 200, methanol extract of *H. caustica* 200 mg/kg; HC 400, methanol extract of *H. caustica* 400 mg/kg; Reference drug diazepam 1 mg/kg.

Among various significant animal tests, elevated plus maze (EPM) is a popular paradigm due to quick valuation of the possible anxiety modulating responses in mice [57]. The typical EPM tool has two opposite open and two closed arms, whereas the open arena is supposed to be more aversive for the animals, and any anxiolytic agent stimulates the mice to the open arm exploration [58]. However, during the experiment, mice treated with MEHC demonstrated a reduction in anxiety-like behavior by reflecting increased entries to and time spent in the open alleys. As shown in Figure 1A,B, MEHC at 400 mg/kg efficiently boosted the % of time spent (*p* < 0.05) in the open arms (48.05 ± 2.47) and the % of open-arm (*p* < 0.01) entries (44.16 ± 2.38), whereas 200 mg/kg responded with a moderate but significant (*p* < 0.05) anxiolytic effect compared to the control group. In contrast, diazepam (reference drug, at 1 mg/kg, i.p.) treated mice exposed a pronounced escalation (*p* < 0.01) in the % of time spent to and the % of entries in the open arms.

**Figure 2:**
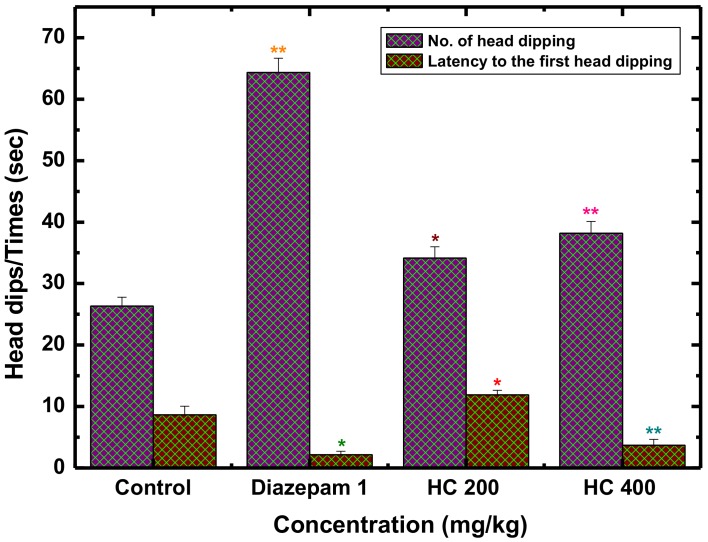
Anxiolytic activity of MEHC and diazepam on hole board test in mice. Values are mean ± S.E.M. (* *p* < 0.05) and (** *p* < 0.01), significantly different from control; ANOVA followed by Dunnett’s test (*n* = 6, per group). MEHC: methanol extract of *Holigarna caustica*; HC 200, methanol extract of *H. caustica* 200 mg/kg; HC 400, methanol extract of *H. caustica* 400 mg/kg; Reference drug diazepam 1 mg/kg.

Similarly, the hole board test (HBT) has been designed to measure the exploratory responses and multiple dimensions of unconditioned behavior of a mouse to an unfamiliar environment [59]. The manifestation of more hole poking (head dipping) tendency indicates high levels of anxiolytic activity, while the hesitancy of visiting hole results as a positive sign of anxiety [60]. In view of this opinion, administration of MEHC at both (200 and 400 mg/kg) doses significantly elevated the exploratory behavior in mice (Figure 2), particularly 400 mg/kg exposed superior (*p* < 0.01) hole poking tendency (38.17 ± 1.92) followed by higher number of head dipping and short duration (*p* < 0.01) of head dipping latency (3.68 ± 0.98). Theses outcomes revealed a dose response anxiolytic nature of MEHC. Moreover, the positive control diazepam showed an increase in the number of head dips (64.33 ± 2.32) compared with the control group (26.33 ± 1.44).

### 3.2. Locomotor and Exploration Activity

**Figure 3:**
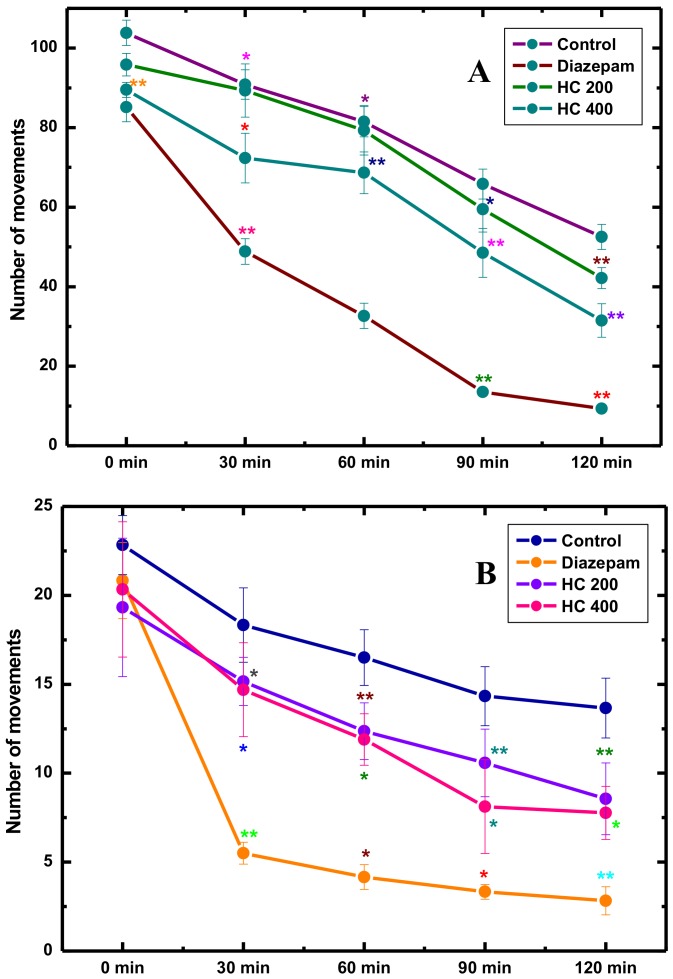
Locomotor and exploratory activities of MEHC and diazepam on open field (**A**) and hole cross (**B**) test in mice. Values are mean ± S.E.M. (* *p* < 0.05) and (** *p* < 0.01), significantly different from control; ANOVA followed by Dunnett’s test (*n* = 6, per group). MEHC: methanol extract of *Holigarna caustica* leaves; HC 200, methanol extract of *H. caustica* 200 mg/kg; HC 400, methanol extract of *H. caustica* 400 mg/kg; Reference drug diazepam 1 mg/kg.

Although promising anxiolytic insights of MEHC have been observed in case of EPM and hole board tests, hence, we further verified the intensity of locomotor and exploratory activities through open field and hole cross assays. The situations of these tests are highly hostile as well as anxiogenic, for which most classical anxiolytic agents are identified from these assessments [61]. In our study, anxiolytics (MEHC at 200 and 400 mg/kg) administration significantly stimulated locomotion and exploration tendency in mice (Figure 3A,B). Utmost agility and CNS (central nervous system) stimulating effects were manifested by the lower dose (200 mg/kg), while exploration and locomotion were almost identical to the control group at all intervals over 120 min. It was reported that anxiolytics with low doses improve the anxiety state by altering motor activity followed by suppressing the muscle relaxation [62]. On the other hand, the reference drug (diazepam, 1 mg/kg) produced tranquility or CNS depressant like activity, particularly, marked decrease in locomotive actions, such as a smaller number of center visiting and sleeping in different locations were also noticed. Importantly, CNS depressant drug like benzodiazepines inhibit excitation and curiosity in mice against the new ambient which leads to decrease their locomotion tendency in consequence [63]. In parallel, a similar pattern of locomotor inhibition was observed following the treatment of higher dose (400 mg/kg) of MEHC. Therefore, it is surmised from all observations that MEHC may have the potential to act as an anxiolytic on CNS which was proved not only by its substantial exploration and locomotor action, but also improved animals’ motor co-ordination activities. 

Basically, the neurobiological mechanism of anxiety is the result of either an imbalance of neurotransmitter (dopamine, GABA, and serotonin) function or dysregulation of glutamatergic, serotonergic, GABA-ergic, and noradrenergic transmission [64]. In our experiment, MEHC may exert anxiolytic actions by modifying the neurotransmitter synthesis and functions. It is supposed that active components of MEHC interact with the neurotransmitter or neuromodulator receptors, which regulate the neuronal communication, stimulate the CNS activity, and improve the function of endocrine system [65]. 

### 3.3. Antidepressant Activity

**Figure 4:**
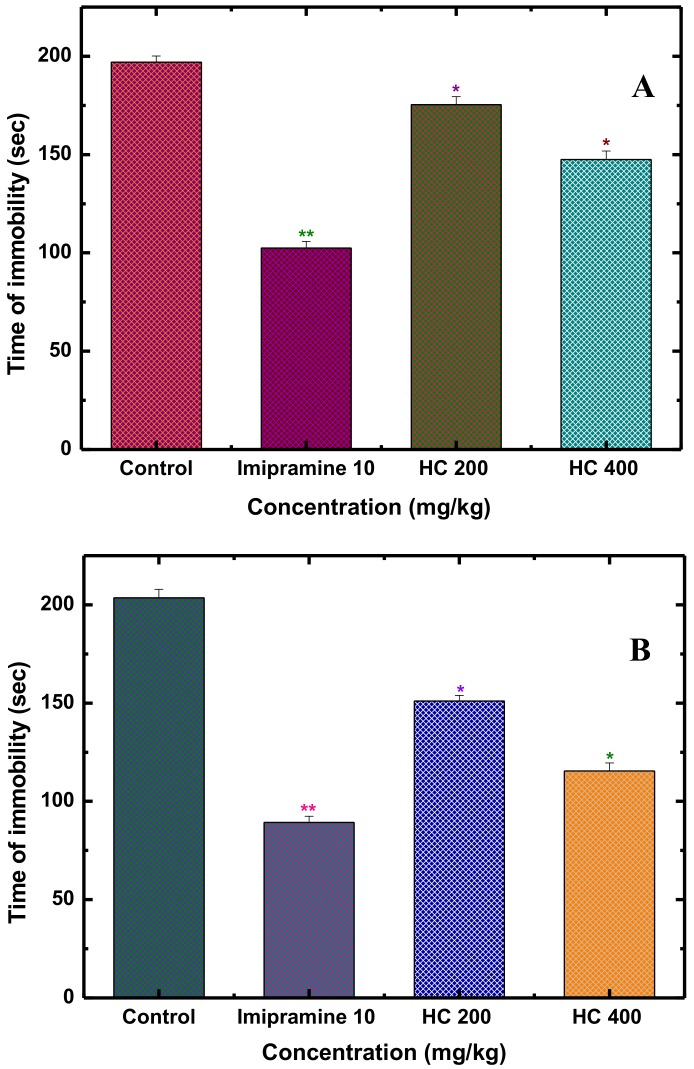
Antidepressant activity of MEHC on forced swimming (**A**) and tail suspension (**B**) tests in mice. Results are expressed in mean ± S.E.M. (* *p* < 0.05) and (** *p* < 0.01), significantly different from control; ANOVA followed by Dunnett’s test (*n* = 6, per group). MEHC: methanol extract of *Holigarna caustica* leaves; HC 200, methanol extract of *H. caustica* 200 mg/kg; HC 400, methanol extract of *H. caustica* 400 mg/kg; Reference drug imipramine hydrochloride (10 mg/kg).

Some potential anxiolytic phyto-medicines, such as *R. rosea* and *C. sativus*, have been verified to have antidepressant effects [66]. This bilateral neuropharmacological action has been known as the “halo effect” whereof once anxiety is cured efficiently, depression may also be de-escalated [67]. In our investigation, alongside the anxiolytic effect, MEHC has also been found to have promising antidepressant potentials, evaluated through force swimming test (FST) and tail suspension test (TST). These tests are very convenient to explore antidepressant-like activity as well as the pathological mechanism of depression [68]. Initially, it was hypothesized that the premise of depression is the impairment of the monoamine transmission system, such as decreasing monoamine production (dopamine, 5-hydroxytryptamine, and norepinephrine) and malfunction of secondary messenger (cyclic AMP or G-proteins) systems [69]. Interestingly, a recent study revealed that the stressful conditions trigger hypothalamic-pituitary-adrenal (HPA) axis which further promotes the neurons in the periventricular nucleus (PVN) to release the corticotropin-releasing factor (CRF) [70]. As we know that depressive symptoms are exposed due to the over activation of HPA axis, resulting dysregulation of CRF, which in that case suppress the adrenocorticotropic hormone (ACTH) response as well as increases the cerebrospinal fluid and plasma cortisol levels [71]. However, successful antidepressant treatment suppresses the stress-induced HPA axis activation, followed by restoring the normal expression and function of CRF [72]. In our study, the control group (1% Tween 80) of mice reflected passive behaviors (a state of behavioral despair e.g., immobility) due to the stressful ambient of FST and TST (Figure 4A,B); while mice treated with MEHC (200 and 400 mg/kg) demonstrated active behaviors (struggling and swimming). In both tests, the noteworthy antidepressant like effect (decreased immobility time) was observed in a dose of 400 mg/kg, which was similar with that of imipramine (10 mg/kg) used as a reference (standard) antidepressant. The evidenced antidepressant activity of MEHC might be either due to inhibition of monoamine reuptake or remarkable suppression of HPA axis over-activation [72].

### 3.4. Histamine-Induced Paw Edema (Anti-Inflammatory Test)

There is substantial evidence that physiological stress stimulates to release various inflammatory mediators (histamine, prostaglandins, cytokines, and leukotrienes) which modifies brain functions, neuroendocrine, and neurotransmission systems, thereby inducing neuro-inflammation and mental disorders [73]. Several reports suggest that cognitive stress triggers mast cells, which are the major sources of histamine, is a peripheral inflammatory mediator and neurotransmitter [74,75]. The activation of four histamine receptors (H_1_R-H_4_R) causes the alteration of pathophysiological and physiological process, where acute expression of H_3_R is associated with neuro-inflammatory disease [76]. In addition, a clinical study revealed that malfunctions of H_3_ receptor are responsible for metabolic syndromes and cognitive impairments such as abnormal behavior and locomotion activities [77]. It is hypothesized that chronic stress induced by FST and TST in mice can stimulate the release of such inflammatory mediators, particularly histamine which contributes to developing depressive and negative moods. Hence, histamine antagonist followed by blocking the release of inflammatory mediators may alleviate the acute inflammatory response at stressed condition. In our neuro-pharmacological analysis, MEHC has been proved to have a promising anxiolytic and antidepressant activity, which in this regard may exhibit the anti-inflammatory response by suppressing the histamine release. To validate this supposition we determined the anti-inflammatory action of MEHC following the histamine challenge. Results revealed that MEHC at both doses (200 and 400 mg/kg) significantly suppressed the inflammatory response (confirmed by paw edema reduction) induced by the sub-planter injection of histamine (Table 1). The stronger inhibitory actions at all hourly intervals over 4 h (43.43%, 51.47%, 59.52%, and 64.28%) manifested by 400 mg/kg were statistically significant (*p* ˂ 0.001) and comparable with that inhibitory response of diclofenac sodium (42.42%, 60.29%, 66.66%, and 78.57%), used as a reference drug at 10 mg/kg. Theses outcomes provide evidence of potential anti-inflammatory efficacy of MEHC and are in accordance with our previous study where MEHC demonstrated significant anti-nociceptive (effectively suppressed the nociception in both central and peripheral pathways) and anti-inflammatory (inhibited carrageenan-induced swelling) activity [18].

### 3.5. In Vitro Antioxidant Activity

**Figure 5:**
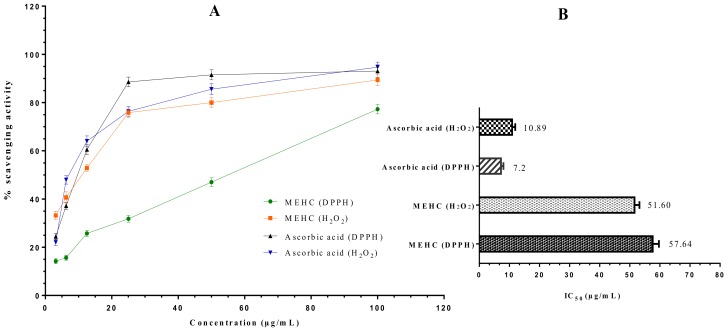
DPPH and H_2_O_2_ free radical scavenging activity of MEHC compared with the reference standard ascorbic acid. (**A**) Percentage of DPPH and H_2_O_2_ free radical scavenging activity by different concentrations of the MEHC and reference standard Ascorbic acid. Values are expressed as mean ± SD (*n* = 3). (**B**) IC_50_ values for DPPH and H_2_O_2_ free radical scavenging activity of MEHC and Ascorbic acid. MEHC refers to methanol extract of *Holigarna caustica* leaves. DPPH: 1,1-diphenyl-2-picrylhydrazyl radical; H_2_O_2_: Hydrogen peroxide.

An imbalance due to over production of cellular reactive oxygen species (ROS) and poor antioxidant defense mechanism makes the brain highly vulnerable and may initiate oxidative stress (OS) [78]. Persistent neuro-inflammation is intimately connected with OS in both physiologically and pathologically [79]. Inflammation cells at a pathological condition generate nitric oxide radical (*NO) and oxygen radical (O_2_^-1^) which subsequently produce a potent oxidizing agent “peroxynitrite anion (ONOO^-1^)”. This anion further disseminates into nitrosonium cations (NO^+1^) and nitroxyl anions (NO^-1^) through DNA fragmentation and lipid peroxidation, thereby inducing OS and physiological dysfunctions [9]. However, the interplay relationship between OS and neuropsychiatric disorders is not surprising, since many elegant studies revealed that cellular OS in the brain can reverse regular neuronal functions, brain activities, and neurotransmissions [80,81]. *Rammal H* et al. 2008 demonstrated a clear interlink between anxiety and OS, where such an imbalance of redox system in mice led to develop recurrent infection, neuro-inflammation, neuro-degeneration, and chronic inflammation [82]. In this regard, antioxidant therapy may ameliorate neuronal functions and OS by inhibiting ROS formation followed by intervening in redox-related signaling pathways. As a potential ROS inhibitor, MEHC manifested significant DPPH and H_2_O_2_ scavenging activities. DPPH is the organic nitrogen radical, whereas brain metabolism produces abundant H_2_O_2_ which is the primary ROS generator in humans. As displayed in Figure 5, free radical scavenging capacity of MEHC was concentration dependent wherein 76.4% of DPPH and 89.6% of H_2_O_2_ free radical scavenging were noted at highest concentration (100 μg/mL), indicating MEHC as a strong antioxidant compared to standard (ascorbic acid scavenged 95.4% DPPH and 96.3% H_2_O_2_ free radical). In addition, 50% inhibitory concentration (IC_50_) values of DPPH and H_2_O_2_ free radical neutralizing activities were found 57.64 μg/mL and 51.60 μg/mL, respectively, while ascorbic acid showed 7.2 μg/mL and 10.89 μg/mL, respectively.

**Figure 6:**
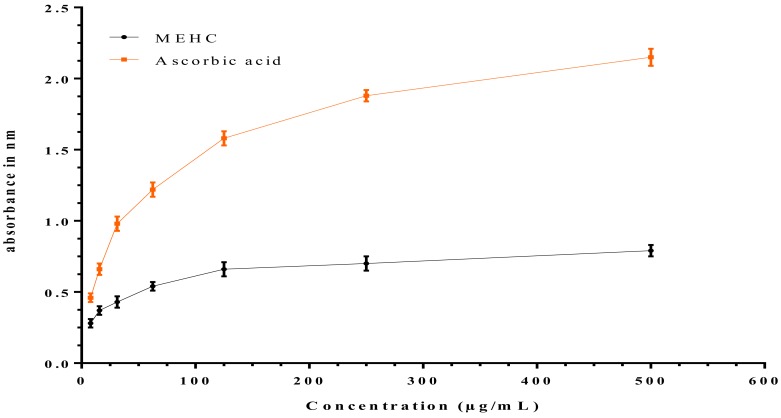
Reducing power capacity of ascorbic acid and MEHC. Values are expressed as mean ± SEM (*n* = 3). MEHC refers to methanol extract of *Holigarna caustica* leaves.

Similarly, redox active metals, including iron (Fe), manganese (Mn), and copper (Cu) are the great contributor of generating various free radicals. Particularly, free iron is oxidized under pathological conditions from Fe^2+^ to Fe^3+^ which may damage normal cellular functions by stimulating higher ROS production [83]. To determine the antioxidant ability of MEHC in reversing Fe^3+^ to Fe^2+^ ions, we conducted ferric reducing antioxidant power assay (FRAP) following the method of potassium ferric cyanide reduction. Result revealed that MEHC as a strong antioxidant reduced Fe^3+^ to Fe^2+^ ions, confirmed by color change from yellow (test solution) to green and prussian blue. The reduction was also monitored by UV-Vis analysis at 700 nm, as increased absorbance is proportional to higher reduction of Fe^3+^ ions. However, similar to ascorbic acid (standard), MEHC exhibited increased absorbance of the reaction mixture with the increasing concentrations of test solution, demonstrating that the MEHC had noteworthy reducing power capacity (Figure 6). 

The current outcomes prove the antioxidant prevalence of MEHC can be considered as a potential antioxidant therapy for the treatment of anxiety and depression related disorders as well as regulating the inflammatory and OS signal transduction. It is reported that dietary and natural antioxidants provide interesting pharmacological interventions in paradigms of anxiety and depression. Mechanistically antioxidants as neuroprotective agents exert comparable antidepressant action as like orthodox antidepressant by elevating the serotonin and norepinephrine level in the synaptic cleft [84]. In contrast, regular supplementation of antioxidants may improve anxiety like behavior by inhibiting ROS and lipid peroxidation following occurrence of OS [9]. However the therapeutics and advantageous actions of antioxidants are due to the presence of polyphenols (secondary phytochemicals) which provide natural defensive mechanism against various diseases [85]. 

### 3.6. Quantitative Phytochemical Analysis

During quantitative phytochemical analysis of MEHC, we have evaluated sufficient amount of polyphenols (Table 2) such as total phenolic content (34.76 ± 1.09) mg gallic acid equivalent/g dried extract, total flavonoid content (48.30 ± 1.62) mg quercetin equivalent/g dried extract, total flavonol content (38.28 ± 0.04) mg quercetin equivalent/g dried extract, and total condensed tannin (112.91 ± 0.25) mg catechin equivalent/g dried extract. Nevertheless, very promising amounts of total antioxidant (307.60 ± 0.36) mg ascorbic acid equivalent/g dried extract were determined in the MEHC.

Polyphenols are the major constituents of herbal medicine having multifaceted biological actions [86], such as phenolic acids which boost up the brain antioxidant status by scavenging wide range of ROS and metal ions, which also inhibit signaling system of inflammation and its key inflammatory mediators, thus improving anxiety and depression [87]. Besides, flavonoids are the class of compounds which suppress oxidative nitrosative-stress, attenuate neuroinflammation, potentiate GABA_A_ receptor-Cl ion channel complex, increase monoamines, serotonin, and dopamine levels in the CNS, and also modulate monoaminergic neurotransmitter [88]. In our previous research, we revealed that MEHC can effectively suppress in both central and peripheral nociception pathways, which prove that the bioactive compounds of MEHC can act on the central nervous system by crossing the blood brain barrier (BBB). In addition, our preliminary phytochemicals screening detected several metabolites, including phenols, alkaloids, terpinoids, steroids, flavonoid, sterols, and saponins [18]. It is reported that such metabolites present in the plant extract possess anxiolytic and antidepressant effect [89,90]. Moreover, GC-MS analysis of MEHC demonstrated 40 potential bioactive constituents, wherein vitamin E and three important phenolic compounds, such as, 3-((4*Z*, 7*Z*)-heptadeca-4, 7-dien-1-yl) phenol, (*Z*)-3-(pentadec-8-en-1-yl) phenol, and 3-pentadecyl-, (Z)-3-(heptadec-10-en-1-yl) phenol were found [18], which might also directly or indirectly be involved in the above mentioned pharmacological activities of MEHC. However, several bioactive compounds of MEHC (from GC-MS data) have been documented based on their biological activities and further analyzed by bioinformatics computational experiments, in order to confirm the pharmacological effects of MEHC on neuropsychiatric disorders. 

### 3.7. In Silico Studies

Our previous qualitative phytochemical study regarding this plant confirmed the presence of numerous phytochemicals such as carbohydrates, alkaloids, flavonoids, terpenoids, proteins, cardiac glycosides, saponins, coumarins, sterols, and steroids. Additionally, our quantitative phytochemical study revealed the significant amount of polyphenols contents in MEHC, as mentioned earlier in Table 2. Moreover, forty phytocompounds were identified in MEHC using GC-MS analysis in our previous study [18]; from them, eleven major bioactive phytocompounds were selected for molecular docking study. In silico molecular docking is the most powerful computational tool which has been broadly used for the prediction of ligand-target interactions and to know the binding modes inside the binding pocket of proteins as well as to understand the possible molecular mechanism of various pharmacological responses [26]. Form this view, an in silico molecular docking study was carried out for a better understanding of the observed pharmacological activities and mechanism of action of MEHC, and an approach had taken to correlate these findings with the experimental results.

Then, these major phytocompounds were docked against five target receptors/enzymes which are primarily responsible for anxiolytic (potassium channel receptor, PDB: 4UUJ), antidepressant (human serotonin receptor, PDB: 5I6X), anti-inflammatory (cyclooxygenase-1 and 2; COX-1, PDB: 2OYE and COX-2, PDB: 3HS5), and antioxidant (xanthine oxidoreductase enzyme, PDB: 1R4U) activities.

**Figure 7:**
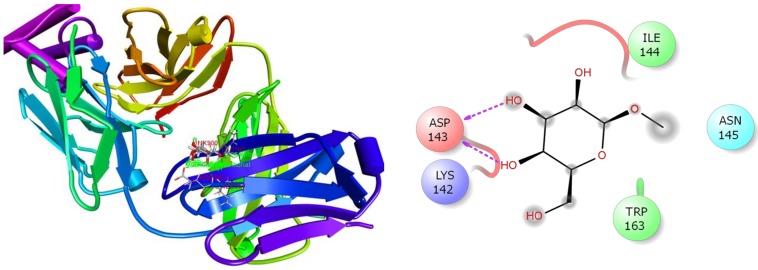
Best ranked poses and 2D interactions of *β*-D-Glucopyranoside, methyl with potassium channel (pdb: 4UUJ) for anxiolytic activity.

In the case of anxiolytic docking study, results of the docking analysis for anxiolytic activity are shown in Table 3. The present study revealed that *β*-D-Glucopyranoside, methyl, and hexadecanoic acid, methyl ester have presented the highest and lowest binding affinity against the potassium channel (PDB: 4UUJ) with a docking score of −3.78 kcal/mol and +3.18 kcal/mol, respectively. The ranking order of docking score for anxiolytic effect is given below: *β*-D-Glucopyranoside, methyl > *β*-Sitosterol acetate > Vitamin E > *α*-Tocospiro A > 2-Pentadecanone, 6,10,14-trimethyl > Elaidic acid > *n*-Hexadecanoic acid > Hexadecanoic acid, methyl ester. The molecular docking study of each compound displayed several binding interactions between the ligands and the target receptor (Table S1). Here, *β*-D-Glucopyranoside, methyl interacted with the potassium channel (PDB: 4UUJ) receptor through five H-bonds to Ile144, Trp163, and Asp143 (three interactions) (Figure 7); *β*-Sitosterol acetate interacted with the same receptor by forming two alkyl interactions with Lys142 and three pi-alkyl interactions with Trp163 (Figure S1); vitamin E showed three alkyl interactions with Lys142, one alkyl interaction with Lys103, two pi-alkyl interactions with Trp163, and one pi-alkyl interaction with Trp173 and Lys142 (Figure S2); *α*-Tocospiro A exhibited five pi-alkyl interactions with Trp163 (Figure S3); 2-Pentadecanone, 6,10,14-trimethyl showed one H-bond with Tyr173, one alkyl interaction with Lys142, two pi-alkyl interactions with Trp163, and one pi-alkyl interaction with Trp173 (Figure S4); elaidic acid exposed one alkyl interaction with Val146 (Figure S5); *n*-Hexadecanoic acid showed two H-bonds with Trp163 and Ile144, one alkyl and pi-alkyl interactions with Lys142 and Trp173 respectively (Figure S6); and hexadecanoic acid, methyl ester did not express any interaction at all (Figure S7). However, neophytadiene, campesterol, and stigmasterol did not dock with the target receptor at all. 

**Figure 8:**
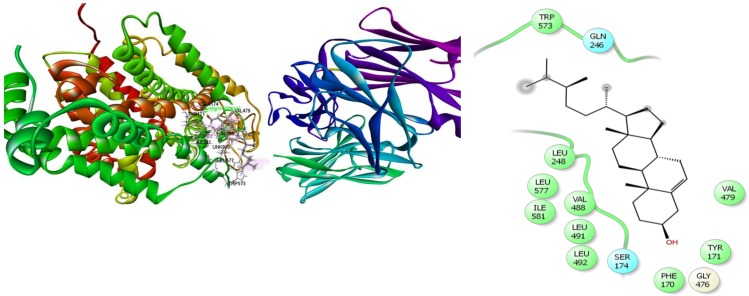
Best ranked poses and 2D interactions of campesterol with human serotonin receptor (pdb: 5I6X) for antidepressant activity.

In the case of antidepressant docking study, campesterol and elaidic acid have shown the highest and lowest binding affinity against human serotonin receptor (PDB: 5I6X) with a docking score of −3.19 kcal/mol and +2.89 kcal/mol, respectively (Table 4). Here, campesterol interacted with the human serotonin receptor (PDB: 5I6X) through one H-bond to Ser174, six alkyl interactions with Val479, Leu577, Ile581, Val479, Val488, Leu492, and five pi-alkyl interactions with Tyr171 and Trp573 (four interactions) (Figure 8) (Table S1); *α*-Tocospiro A interacted with the same receptor by forming one H-bond with Tyr171 and seven alkyl interactions with Leu577, Ile581, Val488, Leu491, Leu492, Ile581, and Leu248 (Figure S8); *β*-Sitosterol acetate demonstrated one H-bond with Gly249, one pi-sigma interaction with Trp573, three alkyl interactions with Leu245 and Leu248 (two interactions), and four pi-alkyl interactions with Trp573 (Figure S9); stigmasterol revealed five alkyl interactions with Leu248 (two interactions) and Val479 (three interactions), and four pi-alkyl interactions with Trp573 (Figure S10); vitamin E showed three alkyl interactions with Ala580, Leu577, Leu248, and two pi-alkyl interactions with Trp573 (Figure S11); 2-Pentadecanone, 6,10,14-trimethyl exposed two H-bonds with Gln246, Trp573, three alkyl interactions with Leu577 and Ile576 (two interactions), and three pi-alkyl interactions with Trp573 (Figure S12); *n*-Hexadecanoic acid exhibited two H-bonds with Gln246, Trp573, and one alkyl interaction with Leu577 (Figure S13); hexadecanoic acid, methyl ester manifested two H-bonds with Gln246, Trp573 (Figure S14); and elaidic acid had shown two alkyl interactions with Val479 and Val488 (Figure S15). 

**Figure 9:**
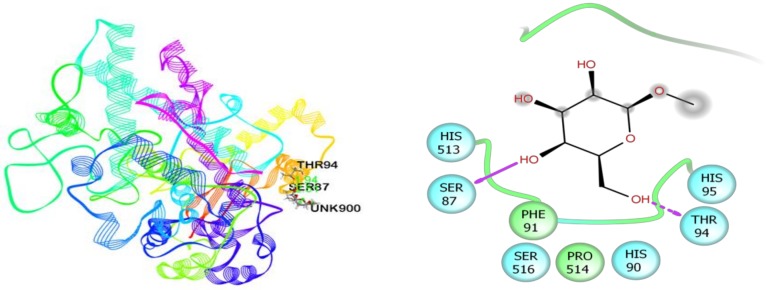
Best ranked poses and 2D interactions of *β*-D-Glucopyranoside, methyl with COX-1 enzyme (pdb: 2OYE) for anti-inflammatory activity.

**Figure 10:**
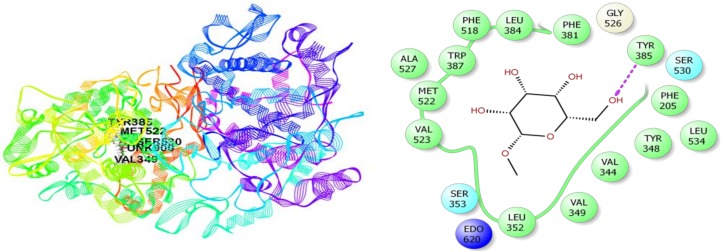
Best ranked poses and 2D interactions of *β*-D-Glucopyranoside, methyl with COX-2 enzyme (pdb: 3HS5) for anti-inflammatory activity.

The selected compounds of MEHC were also docked against COX-1 (PDB: 2OYE) and COX-2 (PDB: 3HS5) enzymes to verify the anti-inflammatory potentials (Table 5). The study disclosed that *β*-D-Glucopyranoside, methyl and elaidic acid have the best binding affinity against both COX-1 and COX-2 enzymes with the highest docking score. Here, *β*-D-Glucopyranoside, methyl interacts with COX-1 (docking score −4.11 kcal/mol) and COX-2 (−5.34 kcal/mol) enzymes through two (Ser87 and Thr94) and five (Tyr385, Ser530, Met522, Ser530, and Val349) hydrogen bonds, respectively (Figure 9 and Figure 10) (Table S2) while elaidic acid demonstrated one pi-alkyl interaction with Phe91 for COX-1 and one hydrogen bond with Met522, three alkyl interactions with Val89, Leu93, Val116, and one pi-alkyl interaction with Tyr115 for COX-2 enzymes (Figures S16 and S17). The remaining compounds did not dock with COX-2 enzyme, but except for the COX-1 enzyme following by the docking score; *β*-Sitosterol acetate (−3.17 kcal/mol), stigmasterol (−2.55 kcal/mol), campesterol (−1.48 kcal/mol), Vitamin E (−1.43 kcal/mol), *α*-Tocospiro A (−1.29 kcal/mol), 2-Pentadecanone, 6,10,14-trimethyl (+1.25 kcal/mol), neophytadiene (+1.47 kcal/mol), hexadecanoic acid, methyl ester (+3.12 kcal/mol), and *n*-Hexadecanoic acid (+3.34 kcal/mol) (Figures S18–S26 ).

**Figure 11:**
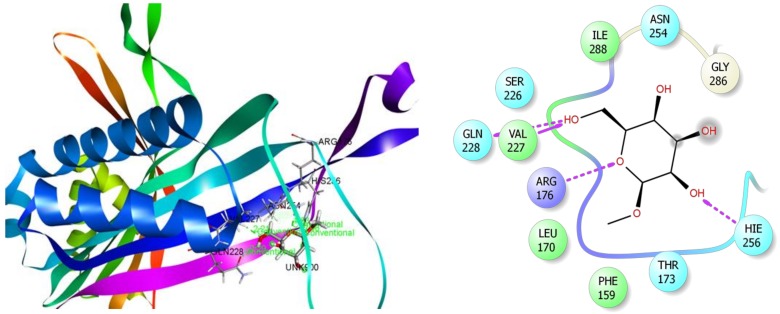
Best ranked poses and 2D interactions of *β*-D-Glucopyranoside, methyl with *xanthine oxidoreductase* (pdb: 1R4U) for antioxidant activity.

Interestingly, the phyto-compound *β*-D-Glucopyranoside, methyl displayed the highest score (−4.81 kcal/mol) during antioxidant docking study (Table 6). This compound also revealed noble binding affinity against anxiolytic and anti-inflammatory enzymes, which indicates the potentiality of this compound. As shown in Figure 11, *β*-D-Glucopyranoside, methyl interacted with the target enzyme xanthine oxidoreductase (PDB ID: 1R4U) through six H-bonds to Arg176, Val227, His256, Gln228, and Asn254 (two interactions) (Table S3). The other ten compounds also demonstrated significant docking score and interaction with xanthine oxidoreductase (Figures S27–S36). Among them, these following compounds manifested good negative score interactions, such as, stigmasterol (docking score: −3.57 kcal/mol; H-bonds interaction: Val227, Asn254; alkyl interaction: Ile288, Leu170; and two pi-alkyl interactions: Phe159); campesterol (docking score: −2.96 kcal/mol; H-bonds: Val227, Asn254, Gln228; alkyl and pi-alkyl: Leu170 and Phe159, respectively); *α*-Tocospiro A (docking score: −2.58 kcal/mol; H-bonds: Gln228, Gly286; alkyl: Ala225, Val227, Ile288; and pi-alkyl: Phe159 and His256); vitamin E (docking score: −1.54 kcal/mol; H-bond: His256; alkyl: Leu170; pi-alkyl: Phe159 and His256; and *β*-Sitosterol acetate (docking score: −1.48 kcal/mol; H-bond: Arg176; alkyl and pi-alkyl: Leu170 and His256), respectively.

From these results, we can conclude that the studied phytocompounds of the MEHC may in part be responsible for the anxiolytic, antidepressant, anti-inflammatory, and antioxidant activities through interactions with these target enzymes or receptors. It has been previously reported that *β*-D-Glucopyranoside, methyl has strong anti-inflammatory effect [91] and it also ameliorates the brain function and epileptic seizure [92]. The compound 2-Pentadecanone, 6,10,14-trimethyl is responsible for anti-inflammatory, cardio-protective, antioxidant, antibacterial, anti-osteoporotic, cytotoxicity, and anticancer activities [93,94]. Hexadecanoic acid, methyl ester possesses antioxidant, hypocholesterolemic, nematicide, and pesticide properties [94] while *n*-Hexadecanoic acid has the antioxidant and anticancer capabilities [94]. Moreover, *β*-Sitosterol acetate is responsible for antioxidant, antipyretic, anti-Inflammatory, anti-arthritic, anti-cancer, anti-diabetic, and antimicrobial activities [95] whereas *α*-Tocospiro A has antituberculasis activity [96,97]. In addition, vitamin E has been reported to have anti-cardiovascular, anti-dementia, anticonvulsant, antioxidant, neuroprotector activities, and improving neurological dysfunction [98,99]. Campesterol has anticancer, antioxidant, hypocholesterolemic, anxiolytic, and antidepressant properties [100,101] whereas stigmasterol has anti-inflammatory and neuroprotective activities, and also responsible for reducing oxidative stress [95,102]. Elaidic acid is responsible for reducing oxidative stress and neurotoxicity [103].

**Figure 12:**
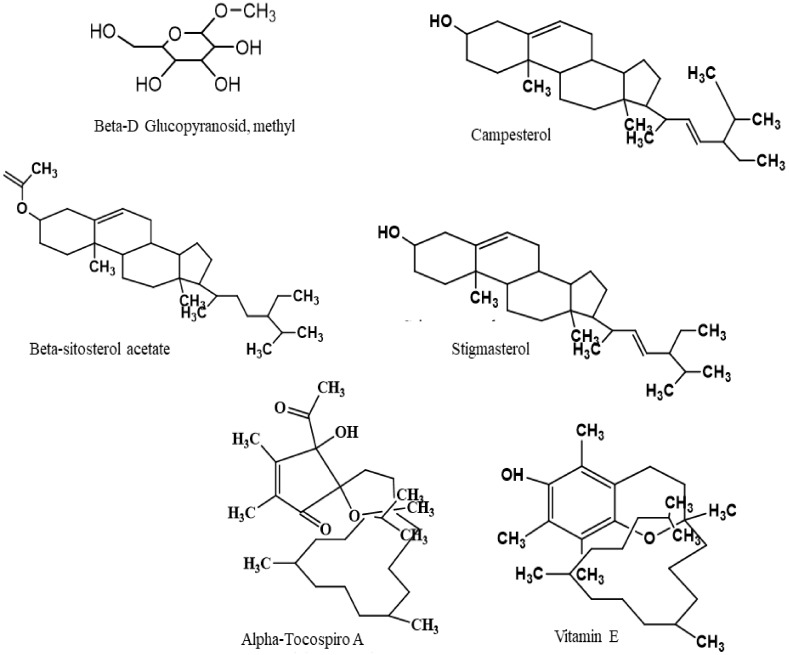
Chemical structures of the major six compounds identified based on the computational study.

According to the highest molecular docking score against potassium channel receptor (anxiolytic), human serotonin receptor (antidepressant), cyclooxygenase enzymes (COX-1 and COX-2) (anti-inflammatory), and xanthine oxidoreductase enzyme (antioxidant), we have selected six phytocompounds (Figure 12) viz. *β*-D Glucopyranoside, methyl, *α*-Tocospiro A, *β*-Sitosterol acetate, campesterol, stigmasterol, and vitamin E in order to search their possible pharmacokinetic or drug-likeliness parameters from draggable point of views and their toxicological properties. 

These types of characterizations are considered to be the most vital step towards the drug discovery process because it does not only save the time of the clinical trial but also, most importantly, saves money [104,105]. In the present study, we used SwissADME, an online tool to calculate the pharmacokinetic properties of the six selected compounds based on Lipinski’s rule of five [106] and Veber’s [107] rules. Here, high oral bioavailability is the most essential factor for the development of new therapeutic agents from bioactive compounds. As stated by the Lipinski’s rule of five, an orally administered drugs/compounds should have a molecular weight < 500 amu, Hydrogen bond acceptor sites < 10, Hydrogen bond donor sites < 5, and lipophilicity value, LogP ≤ 5, whereas Veber et al. proposed that a compound/drug should have the number of rotatable bonds (nRB) ≤ 10 and topological polar surface area (TPSA) value ≤ 140 Å^2^. If any drugs/compounds violate all of these rules, it will not be considered as good oral bioavailability. Results of the present study showed that none of the phytocompounds violate these rules, which indicate good oral bioavailability of the compounds, and these compounds could be considered as possible lead compounds and a good candidate for the development of new drugs (Table 7).

In addition, toxicological properties of the six selected phytocompounds were also predicted using the admetSAR online tool. Results of the present study exhibited that none of the compounds posed a risk of ames toxicity, carcinogenicity, acute oral toxicity, and weak rat acute toxicity (Table 8). As a result, all six phytocompounds could be considered for promising drug candidates with good oral bioavailability through further extensive studies that are still necessary, like a clinical trial on animal models. 

Moreover, to support the conclusions of our laboratory studies, we investigated the possible pharmacological activities of selected phytocompounds, utilizing the structure-based biological activity prediction program “Prediction of activity spectra for substances” (PASS). The value of probable activity (Pa) must be higher than probable of inactivity (Pi) and the value of Pa more than 0.7 is considered pharmacologically potential compound (Table 9). However, the predicted biological activities of all selected phytocompounds were in favor with our laboratory investigations, wherein Pa values of *β*-D Glucopyranoside, methyl and Vitamin E were more promising, which suggest the compounds of MEHC have likely pharmacological potentials and possible targets against specific receptor. 

## 4. Conclusions

The accumulating pharmacological evidences propose that MEHC may provide novel healing insights in neuropsychiatric disorders, particularly anxiety and depression. In our investigation, MEHC has been proved to have promising anxiolytic and antidepressant efficacy. Additionally, further evidence of suppressing the release of inflammatory mediator indicates the anti-inflammatory potential of this plant, which in that case may contribute to inhibit neuro-inflammation followed by inflammation induced neurodegenerative diseases. Furthermore, we have evaluated very favorable amounts of quantitative phytochemicals and antioxidant potentials of MEHC, which can play a preventive role in oxidative stress prompted anxiety and depression. Collectively, these outcomes support the folkloric value and popularity of this plant. Moreover, the computational studies of identified bioactive constituents revealed promising binding affinities towards various receptors in molecular docking analysis. The drug-likeliness, safety, toxicological properties, and possible pharmacological activities of these bioactive constituents were in agreement with our laboratory investigations. Our comprehensive analyses suggest that the predominant efficacies of *H. caustica* may be due to the combined actions of secondary phytochemicals, both already documented herein and hypothetically other as-yet unevaluated phytoconstituents. Therefore, *H. caustica* can be considered as a potential candidate for possible therapeutic intervention in neuropsychiatric disorders. However, more intensive researches are necessary, particularly on the purification of the novel bioactive compounds, and to disclose the molecular mechanisms underlying the observed pharmacological effects.

## Figures and Tables

**Table 1:** Anti-inflammatory effect of methanol extract of Holigarna caustica leaves on histamine-induced paw edema.

Treatment (mg/kg)	Paw Volume (mm) (% Inhibition)			
	1 h	2 h	3 h	4 h
Control	0.454 ± 0.010	0.392 ± 0.012	0.340 ± 0.007	0.312 ± 0.008
RSD 10	0.350 ± 0.004 ***	0.290 ± 0.007 ***	0.264 ± 0.010 ***	0.248 ± 0.012 ***
	(42.42)	(60.29)	(66.66)	(78.57)
MEHC 200	0.398 ± 0.006 ***	0.340 ± 0.010 **	0.298 ± 0.006 *	0.288 ± 0.005
	(29.29)	(39.70)	(52.38)	(46.42)
MEHC 400	0.342 ± 0.005 ***	0.296 ± 0.005 ***	0.264 ± 0.010 ***	0.250 ± 0.010 ***
	(43.43)	(51.47)	(59.52)	(64.28)

Each value is presented as mean ± SEM (*n* = 6); RSD, Reference standard drug, Diclofenac sodium 10 mg/kg; MEHC refers to methanol extract of *Holigarna caustica* leaves. * *p* ˂ 0.05, ** *p* ˂ 0.01, and *** *p* ˂ 0.001 compared with the control group (Dunnett’s Test).

**Table 2:** Total phenolic, flavonoid, flavonol, condensed tannins contents, and total antioxidant capacity of the methanol extract of *Holigarna caustica* leaves.

Tested Sample	Phenolic Content (mg GAE/g Dried Extract)	Flavonoid Content (mg QE/g Dried Extract)	Flavonol Content (mg QE/g Dried Extract)	Condensed Tannins Content (mg CAE/g Dried Extract)	Total Antioxidant Capacity (mg AA/g Dried Extract)
MEHC	34.76 ± 1.09	48.30 ± 1.62	38.28 ± 0.04	112.91 ± 0.25	307.60 ± 0.36

Each value in the table is represented as mean ± SEM (*n* = 3). MEHC refers to methanol extract of *Holigarna caustica* leaves; GAE, gallic acid equivalent; QE, quercetin equivalent; CAE, catechin equivalent; AA refers to ascorbic acid.

**Table 3:** Docking score of the identified compounds with potassium channel receptor (pdb: 4UUJ) for anxiolytic activity. Bold indicate the highest docking score.

Compounds	Docking Score (kcal/mol)	Glide e Model (kcal/mol)	Glide Energy (kcal/mol)
*β*-D-Glucopyranoside, methyl	**−3.78**	−20.33	−18.76
Neophytadiene	-	-	-
2-Pentadecanone, 6,10,14-trimethyl	+0.904	−1.97	−3.30
Hexadecanoic acid, methyl ester	+3.184	−5.15	−10.22
*n*-Hexadecanoic acid	+2.966	−13.37	−17.45
*α*-Tocospiro A	−1.086	−19.61	−20.37
*β*-Sitosterol acetate	−1.827	−19.12	−18.85
Vitamin E	−1.13	−18.08	−18.13
Campesterol	-	-	-
Stigmasterol	-	-	-
Elaidic acid	+2.847	−12.307	−16.48

**Table 4:** Docking score of the identified compounds with human serotonin receptor (pdb: 5I6X) for antidepressant activity. Bold indicate the highest docking score.

Compounds	Docking Score (kcal/mol)	Glide e Model (kcal/mol)	Glide Energy (kcal/mol)
*β*-D-Glucopyranoside, methyl	-	-	-
Neophytadiene	-	-	-
2-Pentadecanone, 6,10,14-trimethyl	+0.009	−19.07	−19.22
Hexadecanoic acid, methyl ester	+2.082	−14.88	−17.09
*n*-Hexadecanoic acid	+2.077	−14.61	−17.99
*α*-Tocospiro A	−2.176	−13.95	−14.51
*β*-Sitosterol acetate	−1.908	−16.89	−17.01
Vitamin E	−1.44	−17.46	−16.76
Campesterol	**−3.199**	−17.20	−16.38
Stigmasterol	−1.589	−8.98	−8.79
Elaidic acid	+2.894	−7.66	−11.696

**Table 5:** Docking score of the identified compounds with COX-1 and COX-2 enzymes for anti-inflammatory activity. Bold indicate the highest docking score.

Compounds	COX-1 (PDB: 2OYE)			COX-2 (PDB: 3HS5)		
	Docking Score (kcal/mol)	Glide e Model (kcal/mol)	Glide Energy (kcal/mol)	Docking Score (kcal/mol)	Glide e Model (kcal/mol)	Glide Energy (kcal/mol)
*β*-D-Glucopyranoside, methyl	**−4.114**	−20.449	−19.099	**−5.34**	−27.901	−24.576
Neophytadiene	+1.476	−11.109	−13.076	-	-	-
2-Pentadecanone, 6,10,14-trimethyl	+1.255	−11.866	−12.886	-	-	-
Hexadecanoic acid, methyl ester	+3.12	−9.837	−12.248	-	-	-
*n*-Hexadecanoic acid	+3.34	−9.918	−12.866	-	-	-
*α*-Tocospiro A	−1.298	−12.789	−12.481	-	-	-
*β*-Sitosterol acetate	−3.178	−19.020	−18.138	-	-	-
Vitamin E	−1.433	−11.413	−10.205	-	-	-
Campesterol	−1.488	−12.445	−12.152	-	-	-
Stigmasterol	−2.556	−16.728	−15.921	-	-	-
Elaidic acid	+2.171	−12.586	−15.874	+0.842	−0.529	−2.204

**Table 6:** Docking score of the identified compounds against *xanthine oxidoreductase* (pdb: 1R4U) for antioxidant activity. Bold indicate the highest docking score.

Compounds	Docking Score (kcal/mol)	Glide e Model (kcal/mol)	Glide Energy (kcal/mol)
*β*-D-Glucopyranoside, methyl	**−4.814**	−32.01	−24.61
Neophytadiene	+2.376	−10.10	−12.78
2-Pentadecanone, 6,10,14-trimethyl	+0.965	−13.3	−14.89
Hexadecanoic acid, methyl ester	+3.362	−9.51	−13.84
*n*-Hexadecanoic acid	+2.812	−16.01	−20.80
*α*-Tocospiro A	−2.584	−31.58	−28.27
*β*-Sitosterol acetate	−1.482	−19.16	−18.37
Vitamin E	−1.544	−22.84	−22.42
Campesterol	−2.968	−21.60	−20.87
Stigmasterol	−3.576	−22.08	−21.61
Elaidic acid	+1.704	−18.825	−22.223

**Table 7:** Physicochemical properties of the selected compounds in MEHC for good oral bioavailability.

Compound	Lipinski Rules				Lipinski’s Violations	Veber Rules	
	MW	HBA	HBD	Log P		nRB	TPSA
	< 500	< 10	< 5	≤ 5	≤ 1	≤ 10	≤ 140
*β*-D Glucopyranoside, methyl	194.18	6	4	−1.64	0	2	99.38
*α*-Tocospiro A	462.70	4	1	6.37	1	13	63.60
*β*-Sitosterol acetate	456.74	2	0	7.63	1	8	26.30
Campesterol	400.68	1	1	6.90	1	5	20.23
Stigmasterol	412.69	1	1	6.96	1	5	20.23
Vitamin E	430.71	2	1	8.27	1	12	29.46

MW, Molecular weight (g/mol); HBA, Hydrogen bond acceptor; HBD, Hydrogen bond donor; Log P, Lipophilicity; nRB: number of rotatable bond; TPSA: topological polar surface area.

**Table 8:** Toxicological properties of the selected compounds in MEHC.

Parameters	Compounds					
	*β*-D Glucopyranoside, Methyl	*α*-Tocospiro A	*β*-Sitosterol Acetate	Campesterol	Stigmasterol	Vitamin E
Ames toxicity	NAT	NAT	NAT	NAT	NAT	NAT
Carcinogens	NC	NC	NC	NC	NC	NC
Acute oral toxicity	III	III	III	I	I	III
Rat Acute Toxicity	1.1350	2.7917	2.0248	2.8078	2.6561	2.1598

NAT, Non Ames toxic; NC, Non-carcinogenic. Category-I means (LD_50_ ≤ 50 mg/kg) and Category-III (500 mg/kg > LD_50_ < 5000 mg/kg).

**Table 9:** Biological activities predicted for *Ophiorrhiza rugosa* major compounds by Prediction of activity spectra for substances (PASS) online.

Compound Name	Biological Properties Predicted by PASS Online	Pa	Pi
*β*-D Glucopyranoside, methyl	GABA aminotransferase inhibitor	0.908	0.002
	Histamine release inhibitor	0.817	0.002
	Free radical scavenger	0.674	0.004
	Lipid peroxidase inhibitor	0.669	0.006
	Antioxidant	0.667	0.004
*α*-Tocospiro A	Anti-inflammatory	0.896	0.004
	peroxidase inhibitor	0.734	0.009
	Antioxidant	0.640	0.004
	Free radical scavenger	0.444	0.014
	Apoptosis agonist	0.403	0.072
*β*-Sitosterol acetate	Prostaglandin-E2 9-reductase inhibitor	0.946	0.003
	Oxidoreductase inhibitor	0.886	0.003
	Peroxidase substrate	0.632	0.004
	Anti-inflammatory	0.575	0.037
	TNF expression inhibitor	0.356	0.072
Campesterol	Wound healing agent	0.501	0.011
	Anti-parkinsonian, rigidity relieving	0.450	0.012
	Dementia treatment	0.745	0.031
	Nitric oxide scavenger	0.353	0.004
	Immunomodulator	0.341	0.050
Stigmasterol	Oxidoreductase inhibitor	0.933	0.001
	Antitoxic	0.755	0.004
	Anti-inflammatory	0.541	0.045
	Lipid metabolism regulator	0.450	0.068
	Immunostimulant	0.360	0.061
Vitamin E	Lipid peroxidase inhibitor	0.978	0.002
	Antioxidant	0.967	0.002
	Acute neurologic disorders treatment	0.935	0.004
	Reductant	0.924	0.006
	Anti-inflammatory	0.830	0.005

Pa = Probable activity; Pi = Probable inactivity.

## Supplementary Materials

The following are available online at https://www.mdpi.com/2218-273X/10/4/561/s1. Table S1. Binding interactions of the identified compounds with potassium channel (pdb: 4UUJ) and human serotonin receptor (pdb: 5I6X) for anxiolytic and antidepressant activity respectively. Best ranked poses and 2D interactions of *β*-Sitosterol acetate, vitamin E, *α*-Tocospiro A, 2-Pentadecanone, 6,10,14-trimethyl, elaidic acid, *n*-Hexadecanoic acid, hexadecanoic acid, methyl ester with human serotonin receptor (pdb: 5I6X) for antidepressant activity (Figures S1–S7). Best ranked poses and 2D interactions of *α*-Tocospiro A, *β*-Sitosterol acetate, stigmasterol, vitamin E, 2-Pentadecanone, 6,10,14-trimethyl, *n*-Hexadecanoic acid, hexadecanoic acid, methyl ester and elaidic acid, with potassium channel (pdb: 4UUJ) for anxiolytic activity (Figures S8–S15). Table S2. Binding interactions of the selected compounds against COX-1 and COX-2 enzymes for anti-inflammatory activity respectively. Best ranked poses and 2D interactions of elaidic acid, *β*-Sitosterol acetate, stigmasterol, campesterol, vitamin E, *α*-Tocospiro A, 2-Pentadecanone, 6,10,14-trimethyl, neophytadiene, hexadecanoic acid, methyl ester, *n*-Hexadecanoic acid with COX-1 (pdb: 2OYE) and COX-2 (pdb: 3HS5) enzymes for anti-inflammatory activity, respectively (Figures S16–S26). Table S3. Binding interactions of the identified compounds in MEHC with *xanthine oxidoreductase* (pdb: 1R4U) for antioxidant activity. Best ranked poses and 2D interactions of stigmasterol, campesterol, *α*-Tocospiro A, vitamin E, *β*-Sitosterol acetate, 2-Pentadecanone, 6,10,14-trimethyl, elaidic acid, neophytadiene, *n*-Hexadecanoic acid, hexadecanoic acid, methyl ester with *xanthine oxidoreductase* (pdb: 1R4U) for antioxidant activity, respectively (Figures S27–S36).

## Abbreviations

MEHC refers to methanol extract of *Holigarna caustica* leaves	
p.o.:	per oral;
i.p.:	Intraperitoneal;
ANOVA:	Analysis of variance;
BW:	body weight;
SEM:	standard error of mean;
SPSS:	statistical package for social science.
ADME/T:	Absorption, Distribution, Metabolism, Excretion, and Toxicity;
PASS:	Prediction of Activity Spectra for Substances.
