# Supplementary material for: Intervention in Neuropsychiatric Disorders by Suppressing Inflammatory and Oxidative Stress Signal and Exploration of In Silico Studies for Potential Lead Compounds from Holigarna caustica (Dennst.) Oken leaves
Source: Biomolecules. 2020 Apr 6;10(4):561. doi: 10.3390/biom10040561 (PMC7226598; doi:10.3390/biom10040561)
Supplement: Supplementary file 1 [file biomolecules-10-00561-s001.pdf]

biomolecules

Supplementary Materials

Intervention in Neuropsychiatric Disorders by Suppressing Inflammatory and Oxidative Stress Signal and Exploration of In Silico Studies for Potential Lead Compounds from *Holigarna caustica* (Dennst.) Oken leaves

Md. Adnan [1,†], Md. Nazim Uddin Chy [2,3,†], A.T.M. Mostafa Kamal [2,*], Kazi Asfak Ahmed Chowdhury [2], Md. Atiar Rahman [4], A. S. M. Ali Reza [2], Md. Moniruzzaman [5], Satyajit Roy Rony [6], Mst. Samima Nasrin [2], Md. Obyedul Kalam Azad [1], Cheol Ho Park [1] and Young Seok Lim [1,*] and Dong Ha Cho [1,*]

[1] Department of Bio-Health Technology, Kangwon National University, Chuncheon 24341, Korea; mdadnan1991.pharma@gmail.com (M.A.); azadokalam@gmail.com (M.O.K.A.); chpark@kangwon.ac.kr (C.H.P.)
[2] Department of Pharmacy, International Islamic University Chittagong, Chittagong 4318, Bangladesh; nazim107282@gmail.com (M.N.U.C.); ashfak4u_ctg@yahoo.com (K.A.A.C.); alirezaru@gmail.com (A.S.M.A.R.); shathy_ru@yahoo.com (M.S.N.)
[3] Drug Discovery, GUSTO A Research Group, Chittagong 4000, Bangladesh
[4] Department of Biochemistry & Molecular Biology, University of Chittagong, Chittagong-4331, Bangladesh; atiar@cu.ac.bd (M.A.R.)
[5] Designated Reference Institute for Chemical Measurement (DRiCM), Bangladesh Council of Scientific & Industrial Research (BCSIR), Dhaka-1205, Bangladesh; monir.accedu@gmail.com (M.M.)
[6] BCSIR Laboratories, Chittagong, Bangladesh Council of Scientific & Industrial Research (BCSIR), Chittagong-4220, Bangladesh; satyajit_pharm@bcsir.gov.bd (S.R.R.)
† These authors contributed equally to this work
* Correspondence: chodh@kangwon.ac.kr (D.H.C.); potatoschool@kangwon.ac.kr (Y.S.L.); mostafa@pharm.iiuc.ac.bd (A.T.M.M.K.)

Table S1. Binding interactions of the identified compounds with potassium channel (pdb: 4UUJ) and human serotonin receptor (pdb: 5I6X) for anxiolytic and antidepressant activity respectively

Proteins	Ligands	Hydrogen Bond Interactions		Hydrophobic Interactions	
		Amino Acid Residue	Distance (Å)	Amino Acid Residue (Bond)	Distance (Å)
4UUJ	Beta-D-Glucopyranoside, methyl	Ile144	2.97	-	-
		Trp163	3.05		
		Asp143	1.81		
		Asp143	1.85		
		Asp143	3.02		
	Neophytadiene			-	-
	2-Pentadecanone, 6,10,14-trimethyl	Tyr173	1.67	Lys142 (Alkyl)	3.83
				Trp163 (Pi-Alkyl)	4.49
				Trp163 (Pi-Alkyl)	5.06
				Tyr173 (Pi-Alkyl)	5.12
	Hexadecanoic acid, methyl ester	-	-	-	-
	n-Hexadecanoic acid	Trp163	2.18	Lys142 (Alkyl)	4.69
		Ile144	1.97	Trp173 (Pi-Alkyl)	4.89
	.alpha.-Tocospiro A	-	-	Trp163 (Pi-Alkyl)	4.69
				Trp163 (Pi-Alkyl)	4.94
				Trp163 (Pi-Alkyl)	5.31
				Trp163 (Pi-Alkyl)	5.28
				Trp163 (Pi-Alkyl)	4.64
	.beta.-Sitosterol acetate	-	-	Lys142 (Alkyl)	4.28
				Lys142 (Alkyl)	3.78
				Trp163 (Pi-Alkyl)	4.95
				Trp163 (Pi-Alkyl)	4.67
				Trp163 (Pi-Alkyl)	4.52
	Vitamin E	-	-	Lys142 (Alkyl)	5.07
				Lys142 (Alkyl)	3.85
				Lys142 (Alkyl)	4.36
				Lys103 (Alkyl)	5.32
				Trp163 (Pi-Alkyl)	4.75
				Trp163 (Pi-Alkyl)	5.03
				Trp173 (Pi-Alkyl)	4.48
				Lys142 (Pi-Alkyl)	5.28
	Campesterol	-	-	-	-
	Stigmasterol	-	-	-	-
	Elaidic acid	-	-	Val146 (Alkyl)	5.38
	Beta-D-Glucopyranoside, methyl	-	-	-	-
	Neophytadiene	-	-	-	-
	2-Pentadecanone, 6,10,14-trimethyl	Gln246	1.94	Leu577 (Alkyl)	4.56
				Ile576 (Alkyl)	3.21
				Ile576 (Alkyl)	5.18
		Trp573	2.03	Trp573 (Pi-Alkyl)	5.18
				Trp573 (Pi-Alkyl)	4.64
				Trp573 (Pi-Alkyl)	5.34
	Hexadecanoic acid, methyl ester	Gln246	2.02	-	-
		Trp573	1.82		
	n-Hexadecanoic acid	Gln246	2.03	Leu577 (Alkyl)	4.38
		Trp573	1.83		
	.alpha.-Tocospiro A	Tyr171	2.05	Leu577 (Alkyl)	4.52

5I6X				Ile581 (Alkyl)	4.86
				Val488 (Alkyl)	4.71
				Leu491 (Alkyl)	4.66
				Leu492 (Alkyl)	4.35
				Ile581 (Alkyl)	4.43
				Leu248 (Alkyl)	4.53
	.beta.-Sitosterol acetate	Gly249	2.65	Trp573 (Pi-Sigma)	2.81
				Leu248 (Alkyl)	4.81
				Leu248 (Alkyl)	5.18
				Leu245 (Alkyl)	4.81
				Trp573 (Pi-Alkyl)	4.27
				Trp573 (Pi-Alkyl)	4.49
				Trp573 (Pi-Alkyl)	4.78
				Trp573 (Pi-Alkyl)	4.08
	Vitamin E			Ala580 (Alkyl)	3.85
				Leu577 (Alkyl)	4.77
				Leu248 (Alkyl)	4.07
				Trp573 (Pi-Alkyl)	3.99
				Trp573 (Pi-Alkyl)	4.10
	Campesterol	Ser174	2.73	Val479 (Alkyl)	4.12
				Leu577 (Alkyl)	5.22
				Ile581 (Alkyl)	4.14
				Val479 (Alkyl)	4.14
				Val488 (Alkyl)	4.76
				Leu492 (Alkyl)	5.24
				Tyr171 (Pi-Alkyl)	5.44
				Trp573 (Pi-Alkyl)	4.85
				Trp573 (Pi-Alkyl)	3.85
				Trp573 (Pi-Alkyl)	3.70
				Trp573 (Pi-Alkyl)	3.33
	Stigmasterol	-	-	Val479 (Alkyl)	3.43
				Leu248 (Alkyl)	4.05
				Val479 (Alkyl)	4.33
				Leu248 (Alkyl)	4.47
				Val479 (Alkyl)	4.38
				Trp573 (Pi-Alkyl)	4.41
				Trp573 (Pi-Alkyl)	4.40
				Trp573 (Pi-Alkyl)	4.39
				Trp573 (Pi-Alkyl)	3.23
	Elaidic acid	-	-	Val479 (Alkyl)	4.27
				Val488 (Alkyl)	3.75

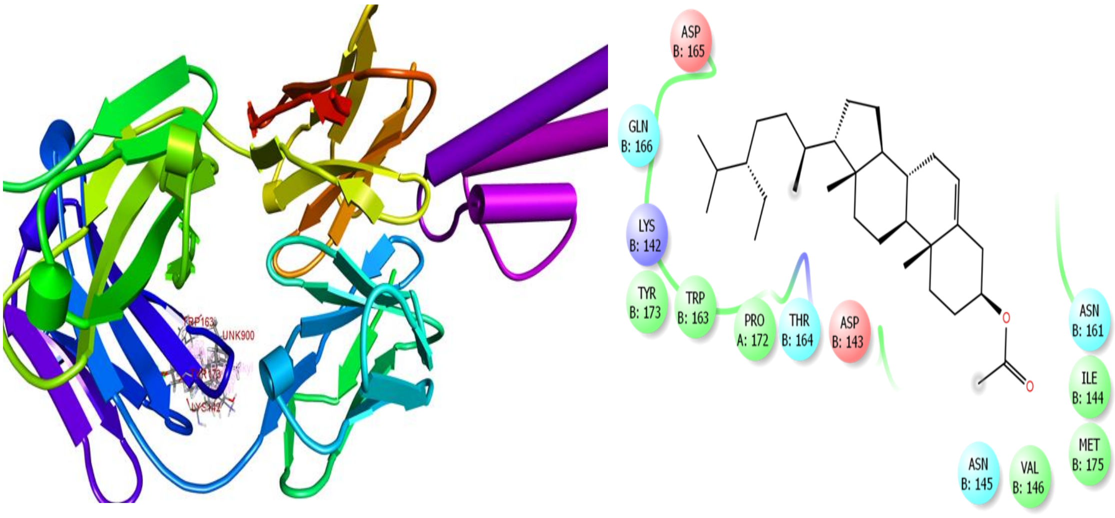

Figure S1. Best ranked poses and 2D interactions of beta.-Sitosterol acetate with potassium channel (pdb: 4UUJ) for anxiolytic activity

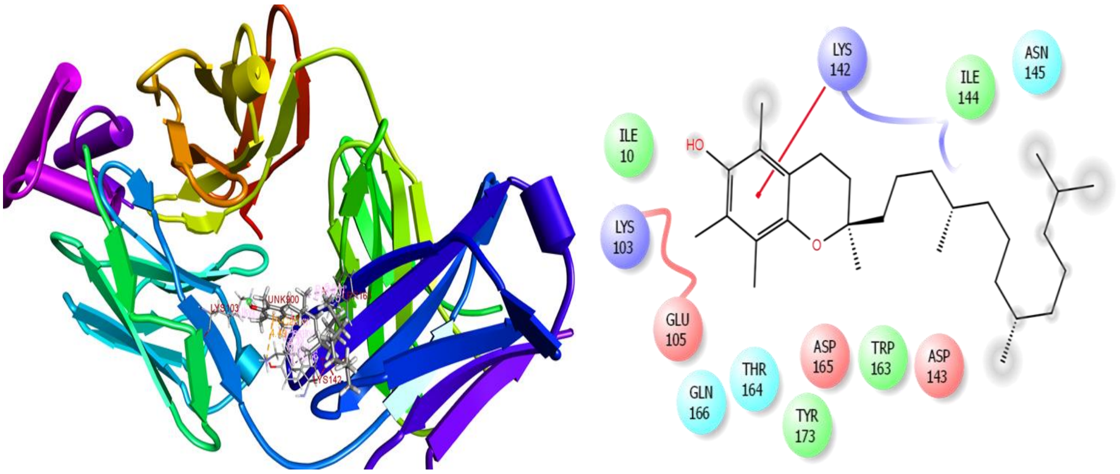

Figure S2. Best ranked poses and 2D interactions of Vitamin E with potassium channel (pdb: 4UUJ) for anxiolytic activity

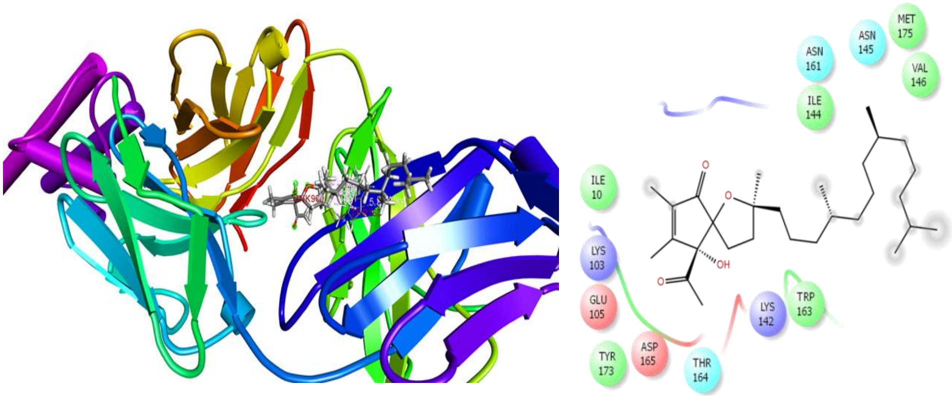

Figure S3. Best ranked poses and 2D interactions of alpha.-Tocospiro A with potassium channel (pdb: 4UUJ) for anxiolytic activity

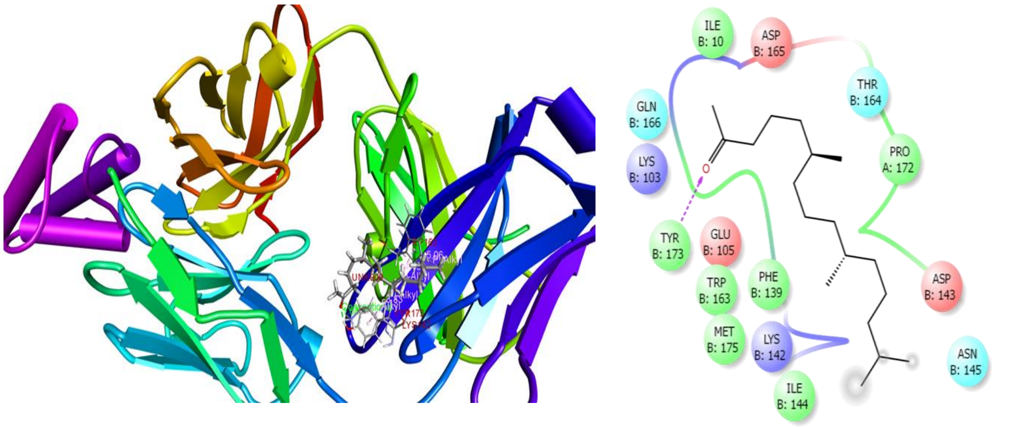

Figure S4. Best ranked poses and 2D interactions of 2-Pentadecanone, 6,10,14-trimethyl with potassium channel (pdb: 4UUJ) for anxiolytic activity

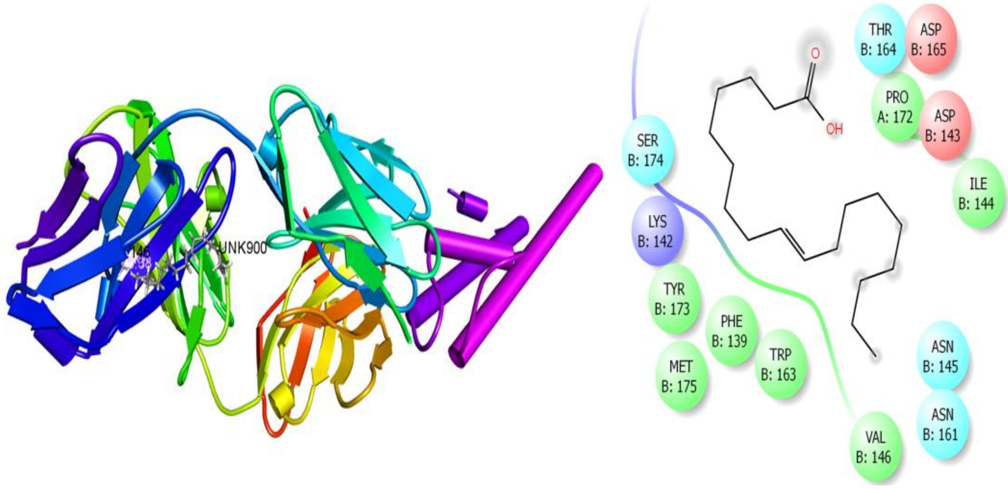

Figure S5. Best ranked poses and 2D interactions of Elaidic acid with potassium channel (pdb: 4UUJ) for anxiolytic activity

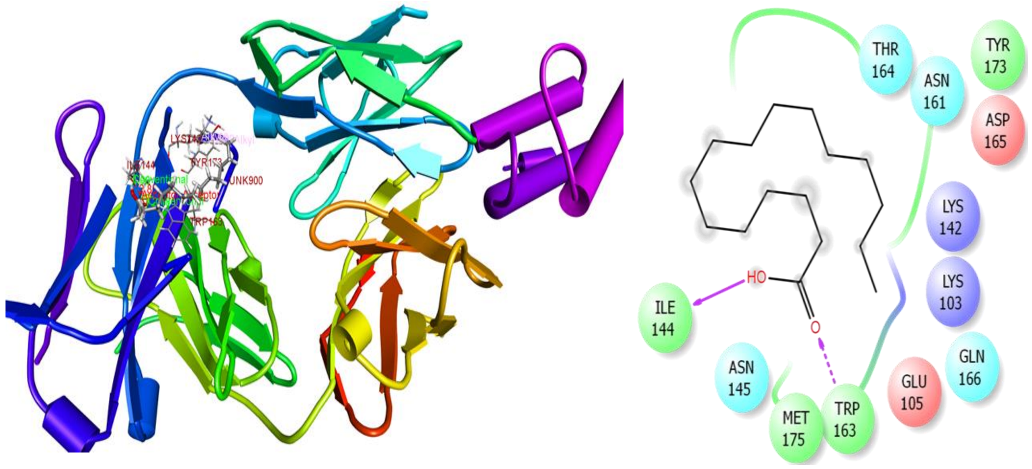

Figure S6. Best ranked poses and 2D interactions of n-Hexadecanoic acid with potassium channel (pdb: 4UUJ) for anxiolytic activity

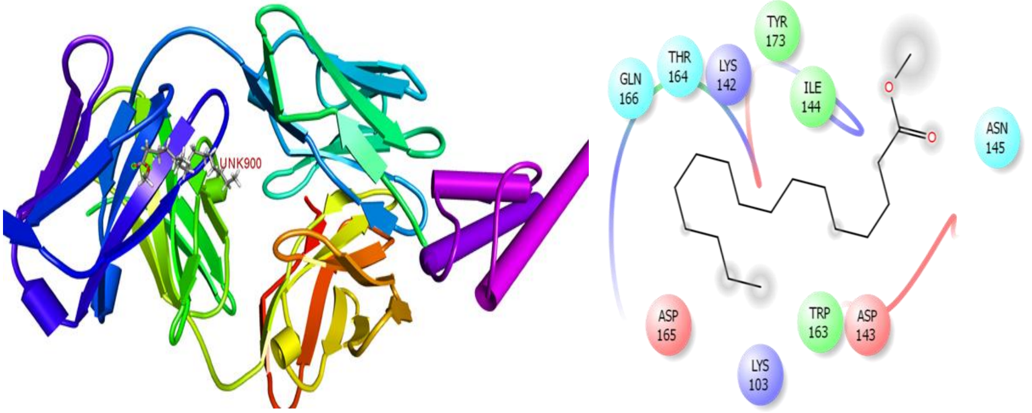

Figure S7. Best ranked poses and 2D interactions of Hexadecanoic acid, methyl ester with potassium channel (pdb: 4UUJ) for anxiolytic activity

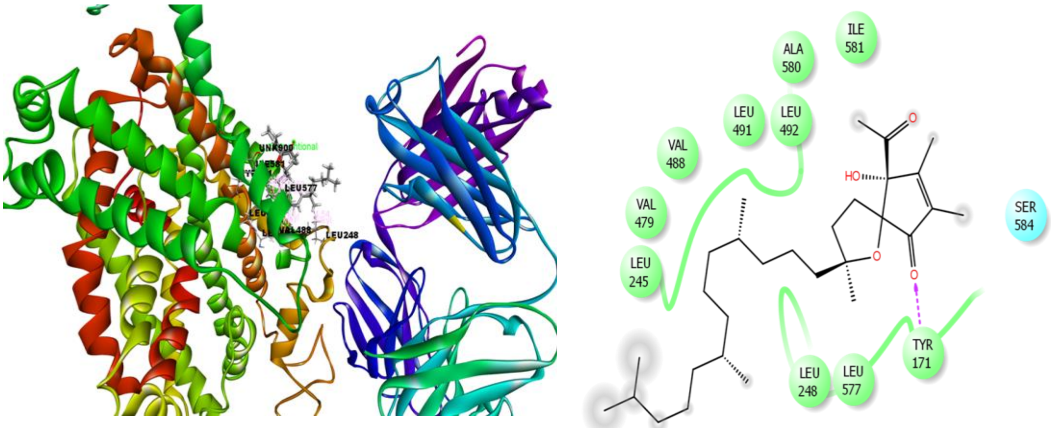

Figure S8. Best ranked poses and 2D interactions of alpha.-Tocospiro A with human serotonin receptor (pdb: 5I6X) for antidepressant activity

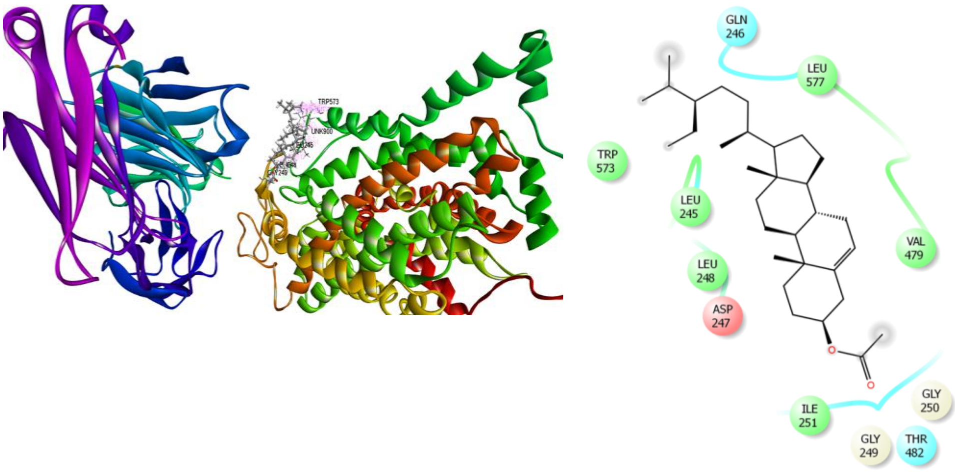

Figure S9. Best ranked poses and 2D interactions of beta.-Sitosterol acetate with human serotonin receptor (pdb: 5I6X) for antidepressant activity

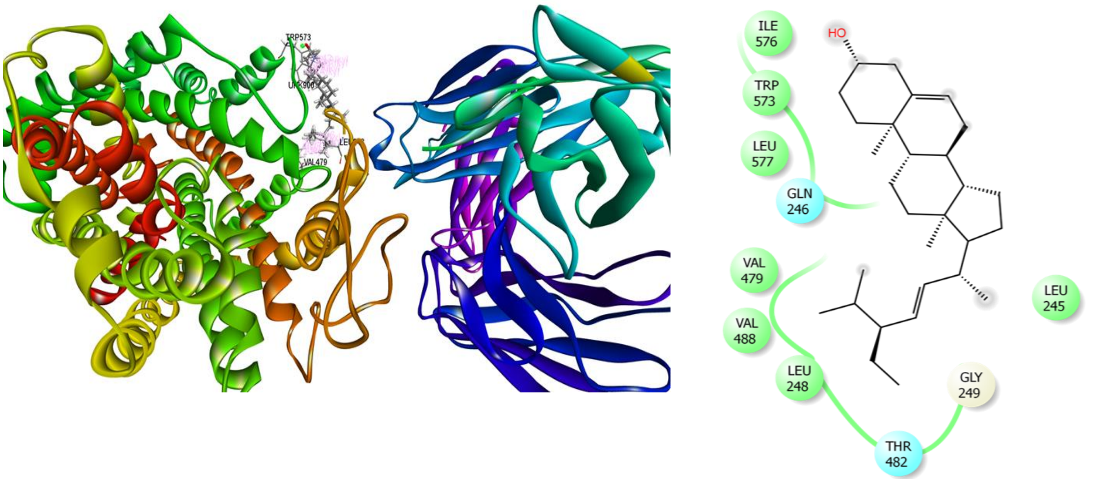

Figure S10. Best ranked poses and 2D interactions of Stigmasterol with human serotonin receptor (pdb: 5I6X) for antidepressant activity

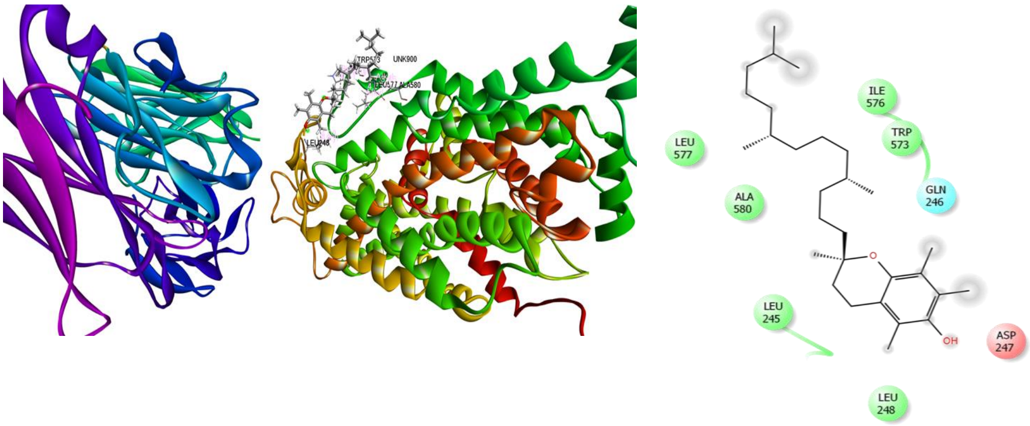

Figure S11. Best ranked poses and 2D interactions of Vitamin E with human serotonin receptor (pdb: 5I6X) for antidepressant activity

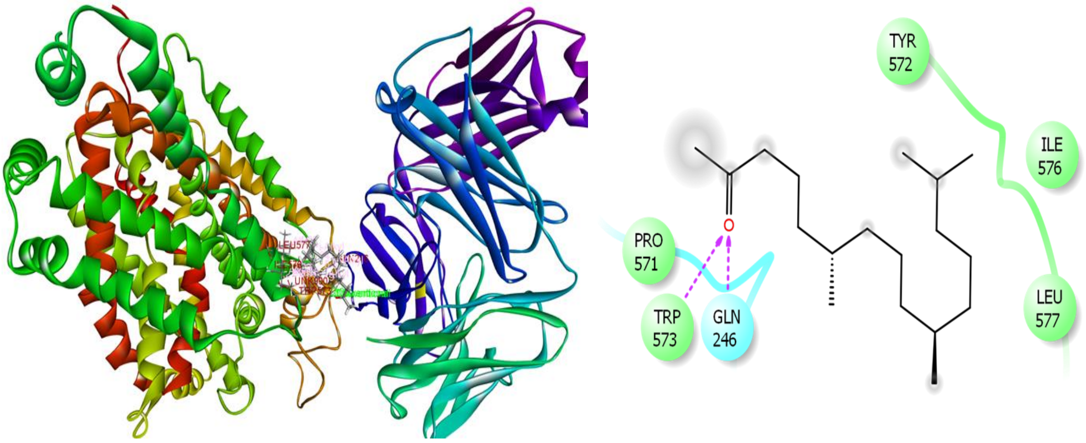

Figure S12. Best ranked poses and 2D interactions of 2-Pentadecanone, 6,10,14-trimethyl with human serotonin receptor (pdb: 5I6X) for antidepressant activity

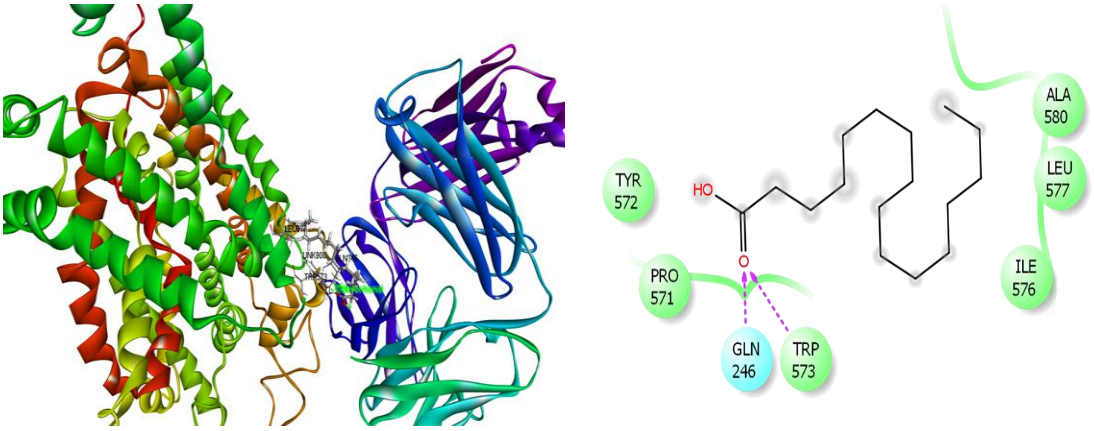

Figure S13. Best ranked poses and 2D interactions of n-Hexadecanoic acid with human serotonin receptor (pdb: 5I6X) for antidepressant activity

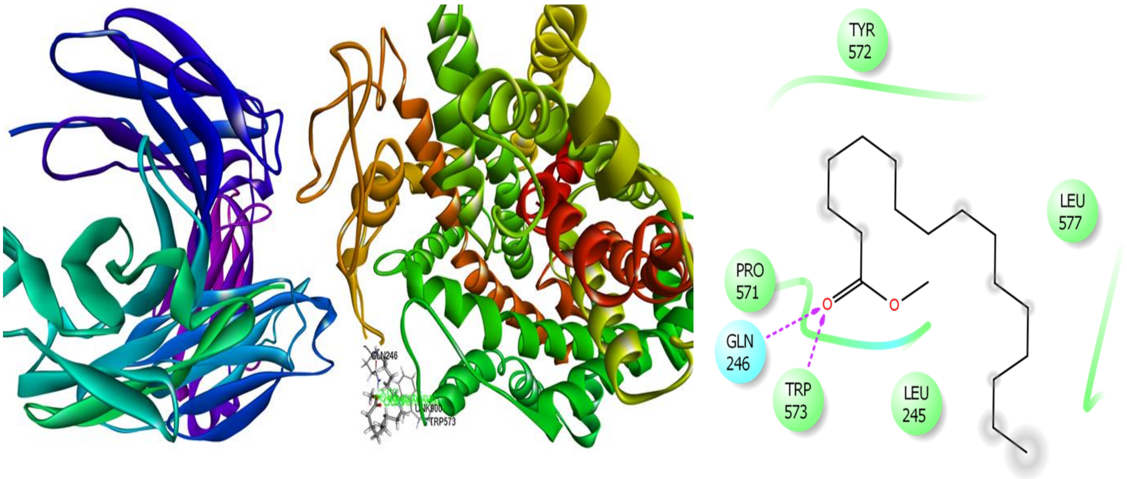

Figure S14. Best ranked poses and 2D interactions of Hexadecanoic acid, methyl ester with human serotonin receptor (pdb: 5I6X) for antidepressant activity

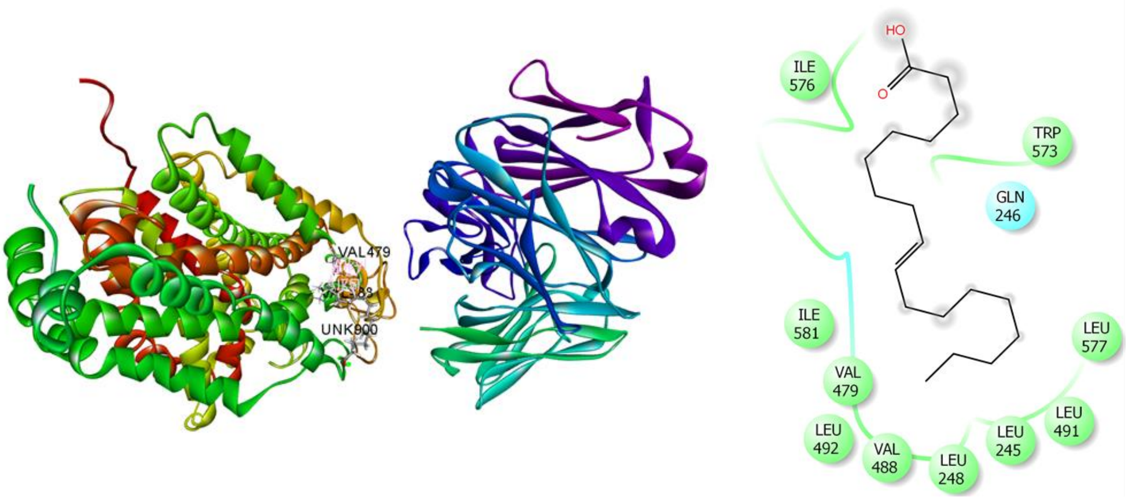

Figure S15. Best ranked poses and 2D interactions of Elaidic acid with human serotonin receptor (pdb: 5I6X) for antidepressant activity

Table S2. Binding interactions of the selected compounds against COX-1 and COX-2 enzymes for anti-inflammatory activity.

Proteins	Ligands	Hydrogen bond interactions		Hydrophobic interactions	
		Amino Acid Residue	Distance (Å)	Amino Acid Residue (bond)	Distance (Å)
COX-1 (PDB: 2OYE)	Beta-D-Glucopyranoside, methyl	Ser87	2.51	-	-
		Thr94	1.93		
	Neophytadiene			Pro514 (Alkyl)	5.06
				Phe88 (Pi-Alkyl)	4.75
				His90 (Pi-Alkyl)	3.86
				Phe91 (Pi-Alkyl)	4.06
				Phe91 (Pi-Alkyl)	4.02
				His95 (Pi-Alkyl)	4.73
				His513 (Pi-Alkyl)	4.47
				His513 (Pi-Alkyl)	5.25
	2-Pentadecanone, 6,10,14-trimethyl	Ser85	2.30	Phe91 (Pi-sigma)	2.46
				Leu92 (Alkyl)	4.78
				Phe91 (Pi-Alkyl)	5.17
	Hexadecanoic acid, methyl ester	Phe88	3.01	-	-
	n-Hexadecanoic acid	Ser85	2.36	Leu92 (Alkyl)	5.41
		Ser87	2.50	Phe91 (Pi-Alkyl)	4.76
		Ser85	3.02		
	.alpha.-Tocospiro A	His513	2.80	Pro514 (Alkyl)	4.17
		Pro514	2.40	Phe91 (Pi-Alkyl)	5.23
				Phe91 (Pi-Alkyl)	4.99
				Phe91 (Pi-Alkyl)	4.61
				His95 (Pi-Alkyl)	5.47
				His95 (Pi-Alkyl)	5.17
	.beta.-Sitosterol acetate	-	-	Pro514 (Alkyl)	5.01
				Pro514 (Alkyl)	5.20
				Pro514 (Alkyl)	3.32
				His90 (Pi-Alkyl)	4.72
				Phe91 (Pi-Alkyl)	5.26
				Phe91 (Pi-Alkyl)	5.45
				Phe91 (Pi-Alkyl)	4.07
				His95 (Pi-Alkyl)	4.77
				His95 (Pi-Alkyl)	4.38
	Vitamin E	His90	3.03	His513 (Pi-Pi Stacked)	5.42
				Phe91 (Pi-Pi T shaped)	5.80
				Pro514 (Alkyl)	3.50
				Pro514 (Alkyl)	3.46
				Phe88 (Pi-Alkyl)	3.90
				His90 (Pi-Alkyl)	4.47
				Phe91 (Pi-Alkyl)	4.66
				Phe91 (Pi-Alkyl)	4.64
				Phe91 (Pi-Alkyl)	4.96
				Pro514 (Pi-Alkyl)	4.86
	Campesterol			Phe88 (Pi-Alkyl)	5.20
				Phe91 (Pi-Alkyl)	5.49
				Phe91 (Pi-Alkyl)	5.04
				Phe91 (Pi-Alkyl)	4.71
				Phe91 (Pi-Alkyl)	4.10
				Phe91 (Pi-Alkyl)	3.87

	Stigmasterol	Cys512	2.91	Lys511 (Alkyl)	4.79
				Pro514 (Alkyl)	4.62
				Phe588 (Pi-Alkyl)	5.08
				Phe91 (Pi-Alkyl)	3.31
				Phe91 (Pi-Alkyl)	3.97
				His513 (Pi-Alkyl)	4.98
				His513 (Pi-Alkyl)	5.13
	Elaidic acid	-	-	Phe91 (Pi-Alkyl)	3.99
COX-2 (PDB: 3HS5)					
	Beta-D-Glucopyranoside, methyl	Tyr385	2.23	-	-
		Ser530	1.81		
		Met522	2.96		
		Ser530	2.62		
		Val349	2.77		
	Neophytadiene	-	-	-	-
	2-Pentadecanone, 6,10,14-trimethyl	-	-	-	-
	Hexadecanoic acid, methyl ester	-	-	-	-
	n-Hexadecanoic acid	-	-	-	-
	.alpha.-Tocospiro A	-	-	-	-
	Vitamin E				
	Campesterol	-	-	-	-
	Stigmasterol	-	-	-	-
	Elaidic acid	Met522	2.07	Val89 (Alkyl)	4.64
				Leu93 (Alkyl)	5.30
				Val116 (Alkyl)	4.45
				Tyr115 (Pi-Alkyl)	4.28

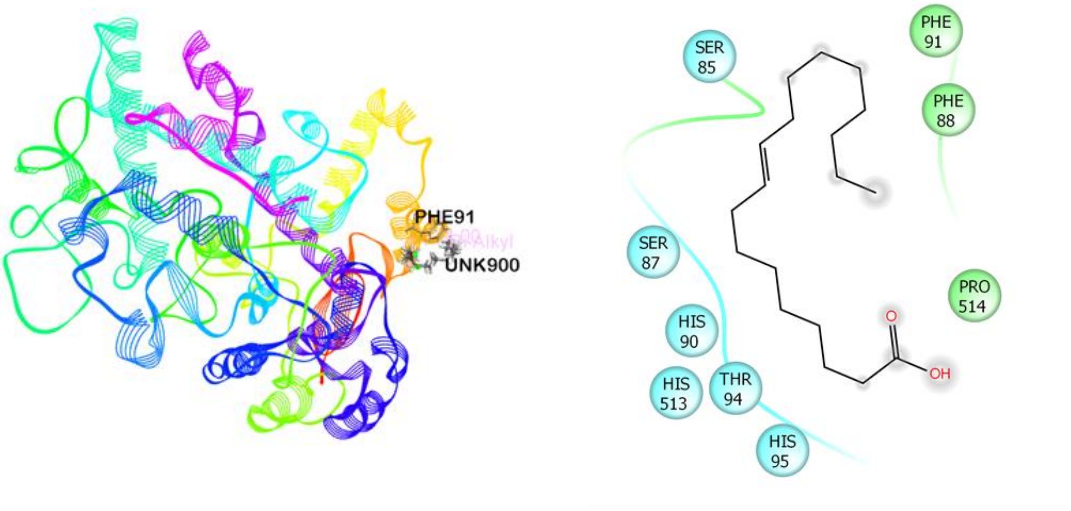

Figure S16. Best ranked poses and 2D interactions of Elaidic acid with COX-1 enzyme (pdb: 2OYE) for anti-inflammatory activity

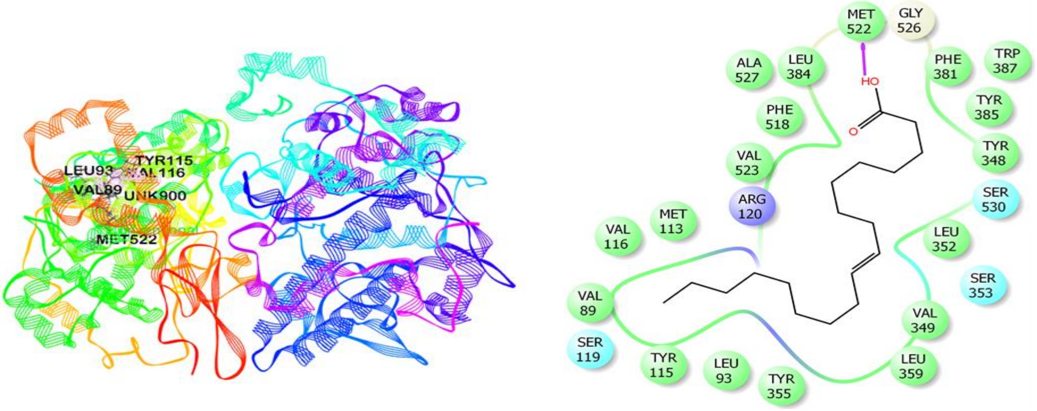

Figure S17. Best ranked poses and 2D interactions of Elaidic acid with COX-2 enzyme (pdb: 3HS5) for anti-inflammatory activity

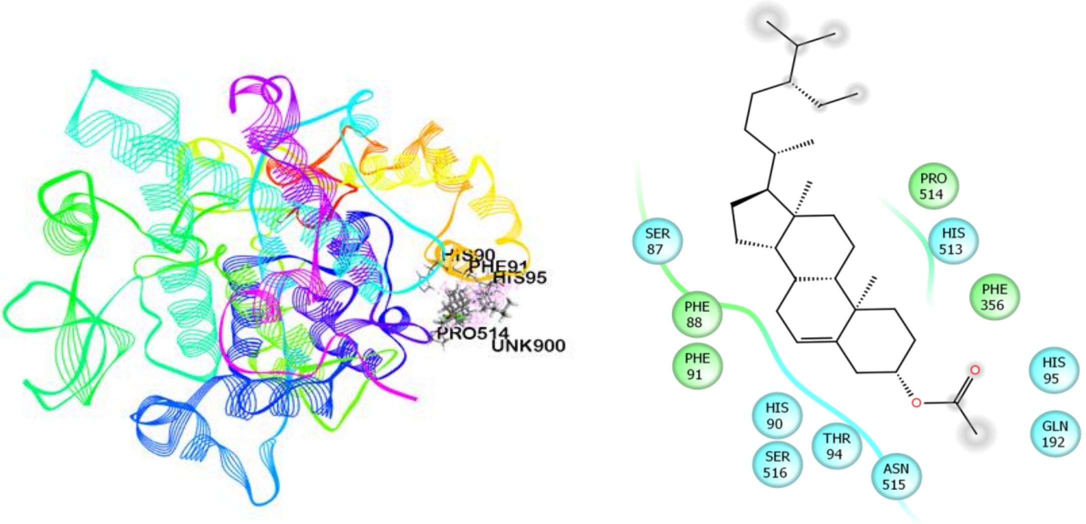

Figure S18. Best ranked poses and 2D interactions of beta.-Sitosterol acetate with COX-1 enzyme (pdb: 2OYE) for anti-inflammatory activity

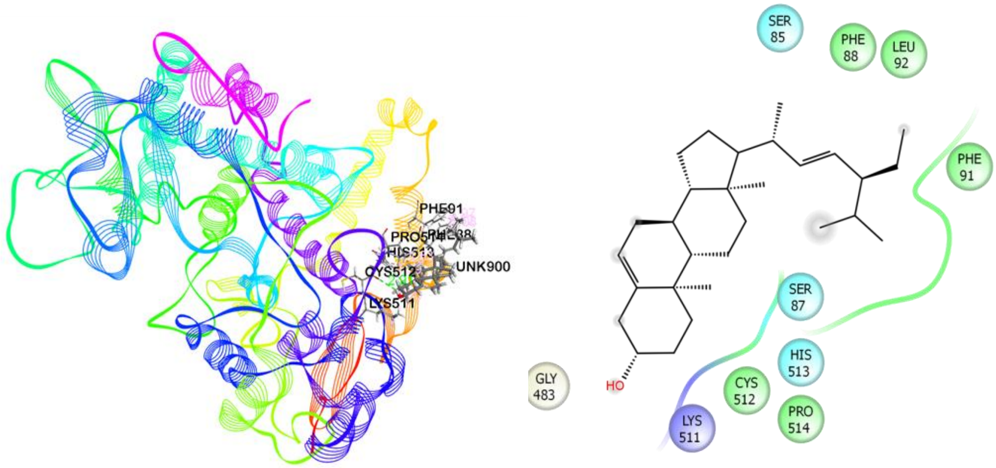

Figure S19. Best ranked poses and 2D interactions of Stigmasterol with COX-1 enzyme (pdb: 2OYE) for anti-inflammatory activity

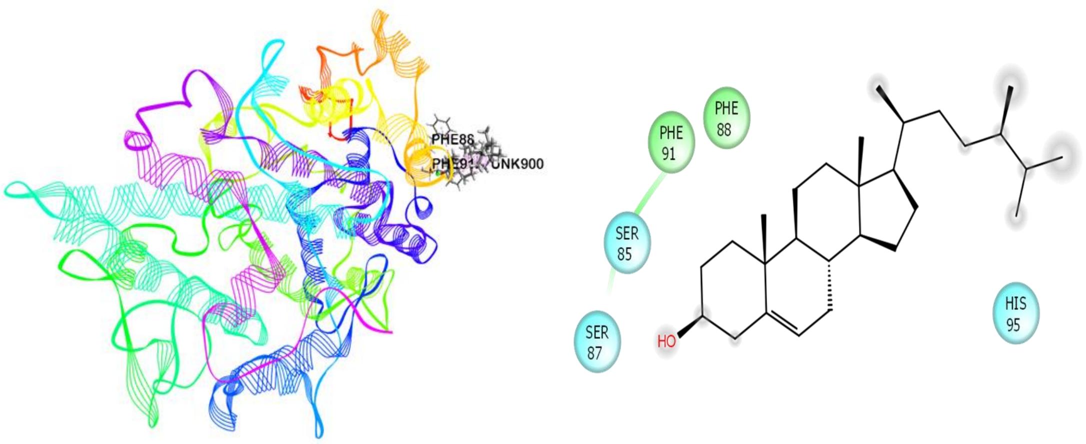

Figure S20. Best ranked poses and 2D interactions of Campesterol with COX-1 enzyme (pdb: 2OYE) for anti-inflammatory activity

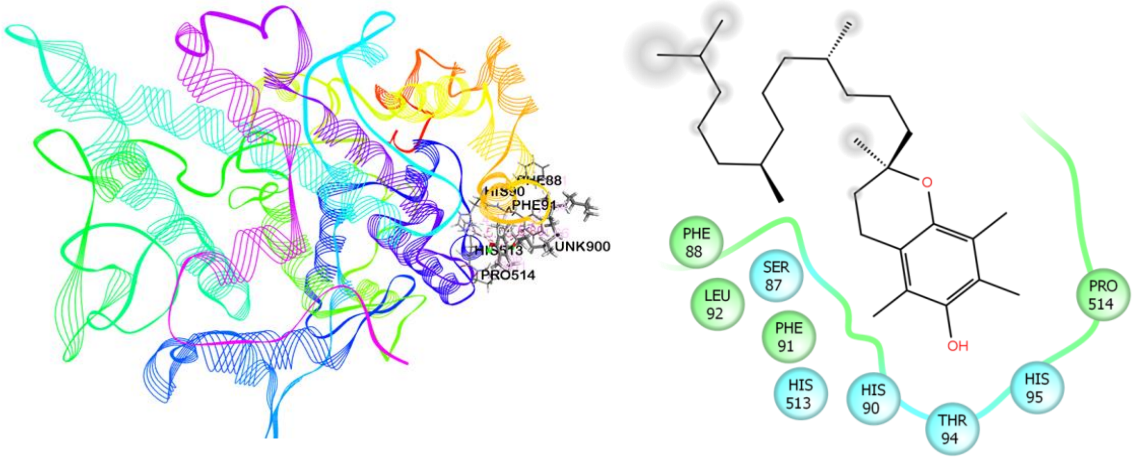

Figure S21. Best ranked poses and 2D interactions of Vitamin E with COX-1 enzyme (pdb: 2OYE) for anti-inflammatory activity

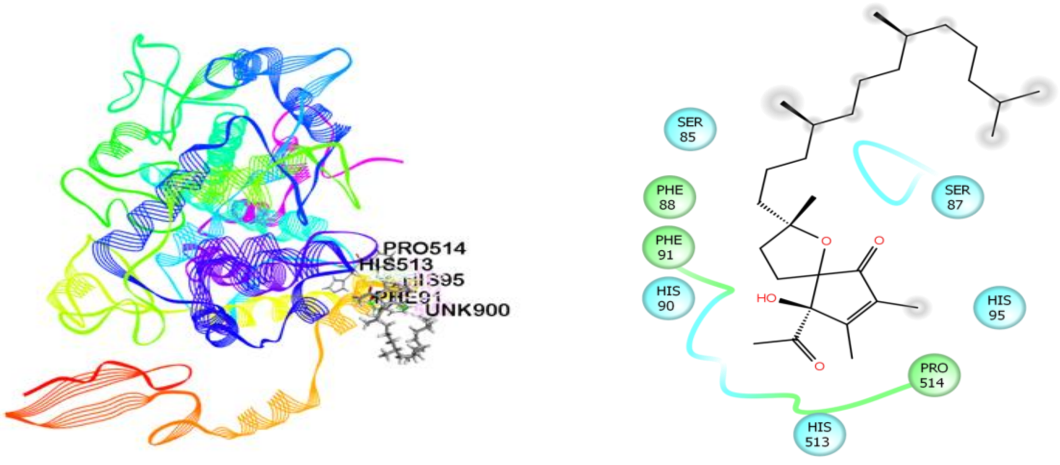

Figure S22. Best ranked poses and 2D interactions of alpha.-Tocospiro A with COX-1 enzyme (pdb: 2OYE) for anti-inflammatory activity

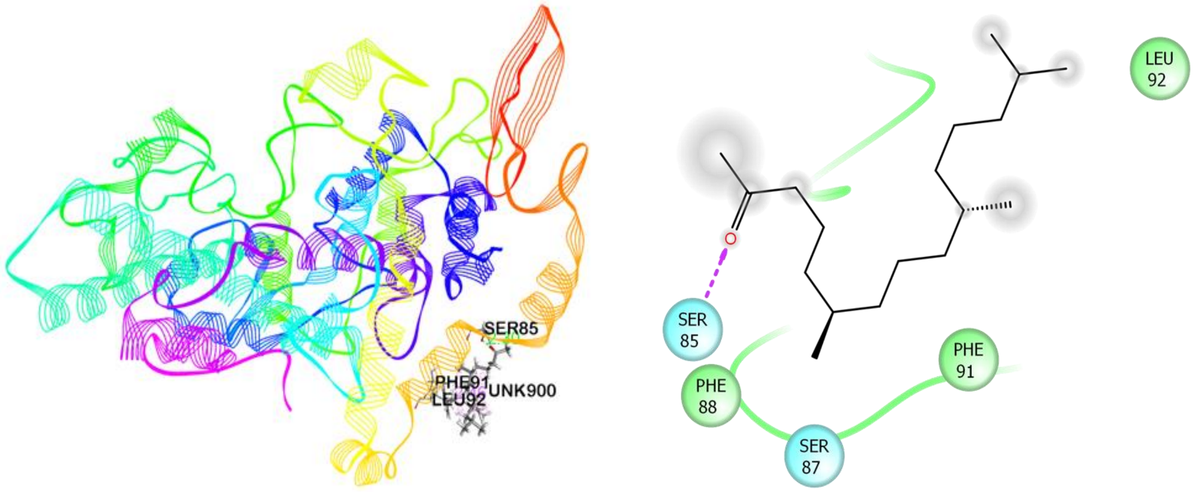

Figure S23. Best ranked poses and 2D interactions of 2-Pentadecanone, 6,10,14-trimethyl with COX-1 enzyme (pdb: 2OYE) for anti-inflammatory activity

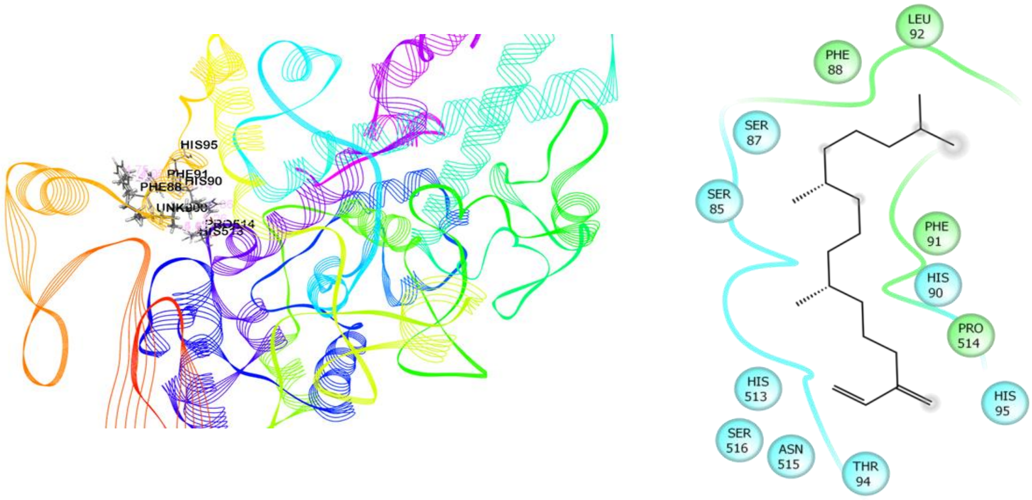

Figure S24. Best ranked poses and 2D interactions of Neophytadiene with COX-1 enzyme (pdb: 2OYE) for anti-inflammatory activity

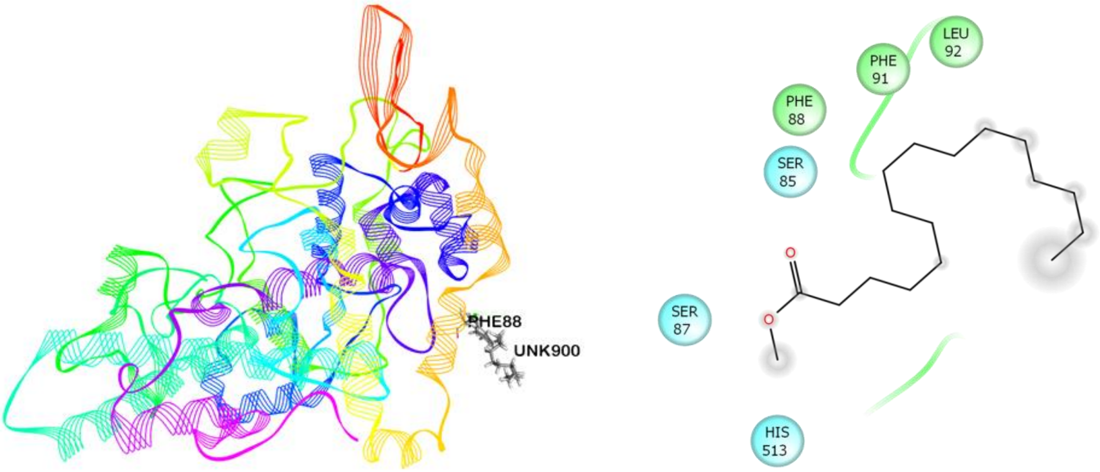

Figure S25. Best ranked poses and 2D interactions of Hexadecanoic acid, methyl ester with COX-1 enzyme (pdb: 2OYE) for anti-inflammatory activity

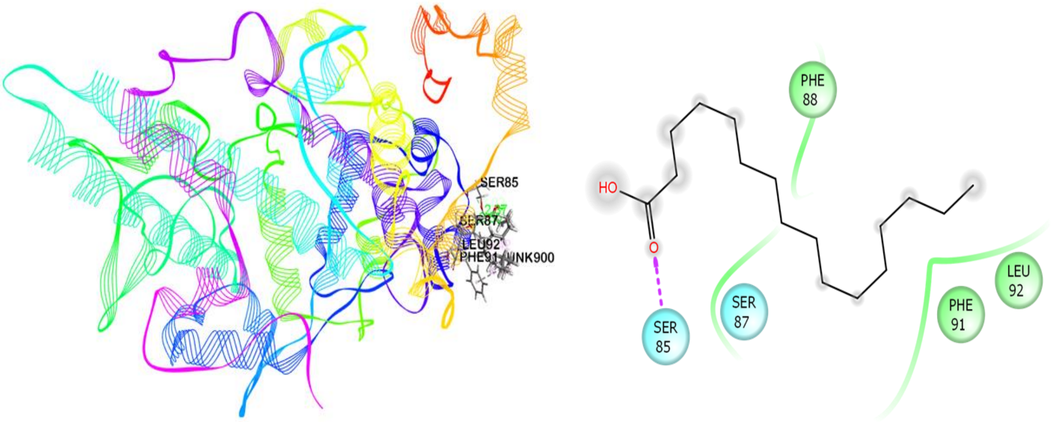

Figure S26. Best ranked poses and 2D interactions of n-Hexadecanoic acid with COX-1 enzyme (pdb: 2OYE) for anti-inflammatory activity

Table S3. Binding interactions of the identified compounds in MEHC with *xanthine oxidoreductase* (pdb: 1R4U) for antioxidant activity.

Compounds	Hydrogen Bond Interactions		Hydrophobic Interactions	
	Amino Acid Residue	Distance (Å)	Amino Acid Residue (bond)	Distance (Å)
Beta-D-Glucopyranoside, methyl	Arg176	1.83	-	-
	Val227	2.20		
	His256	2.16		
	Gln228	2.29		
	Asn254	2.57		
	Asn254	2.31		
Neophytadiene	-	-	Arg176 (Alkyl)	4.42
			Arg176 (Alkyl)	4.17
			Leu170 (Alkyl)	4.33
			His256 (Pi-Alkyl)	4.30
			His256 (Pi-Alkyl)	4.76
			Phe258 (Pi-Alkyl)	5.23
			Phe258 (Pi-Alkyl)	5.49
2-Pentadecanone, 6,10,14-trimethyl	Arg176	1.77	Ile288 (Alkyl)	4.02
			Phe159 (Pi-Alkyl)	4.98
Hexadecanoic acid, methyl ester	Ser226	2.51	Leu170 (Alkyl)	4.26
			Phe159 (Pi-Alkyl)	4.19
n-Hexadecanoic acid	His256	2.24	Phe162 (Pi-Alkyl)	4.73
	Phe159	2.56		
.alpha.-Tocospiro A	Gln228	1.98	Ala225 (Alkyl)	4.18
			Val227 (Alkyl)	4.60
	Gly286	2.28	Ile288 (Alkyl)	5.24
			Phe159 (Pi-Alkyl)	4.45
			His256 (Pi-Alkyl)	4.47
.beta.-Sitosterol acetate	Arg176	2.21	Leu170 (Alkyl)	5.26
			His256 (Pi-Alkyl)	4.83
Vitamin E	His256	2.68	Leu170 (Alkyl)	4.40
			Phe159 (Pi-Alkyl)	3.17
			Phe159 (Pi-Alkyl)	4.96
			His256 (Pi-Alkyl)	4.65
			His256 (Pi-Alkyl)	4.05
Campesterol	Val227	2.36	Leu170 (Alkyl)	4.52
	Asn254	2.38	Leu170 (Alkyl)	4.73
			Phe159 (Pi-Alkyl)	4.43
	Gln228	3.06	Phe159 (Pi-Alkyl)	3.68
Stigmasterol	Val227	2.14	Ile288 (Alkyl)	5.03
	Asn254	2.26	Leu170 (Alkyl)	4.47
			Phe159 (Pi-Alkyl)	4.20
			Phe159 (Pi-Alkyl)	4.05
Elaidic acid	Arg176	2.88	His256 (Pi-Alkyl)	3.85
	Arg176	2.13	Phe258 (Pi-Alkyl)	4.66
	Val227	2.17		

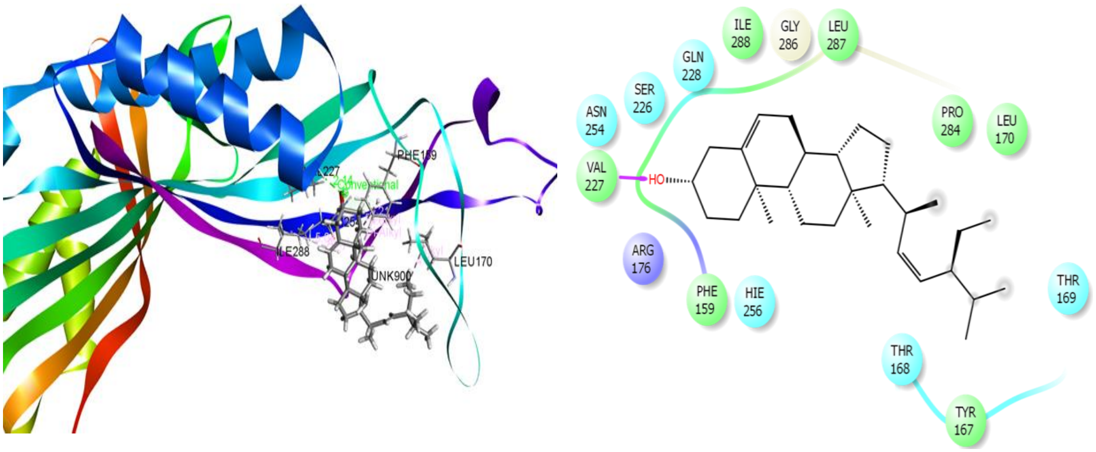

Figure S27. Best ranked poses and 2D interactions of stigmasterol with *xanthine oxidoreductase* (pdb: 1R4U) for antioxidant activity

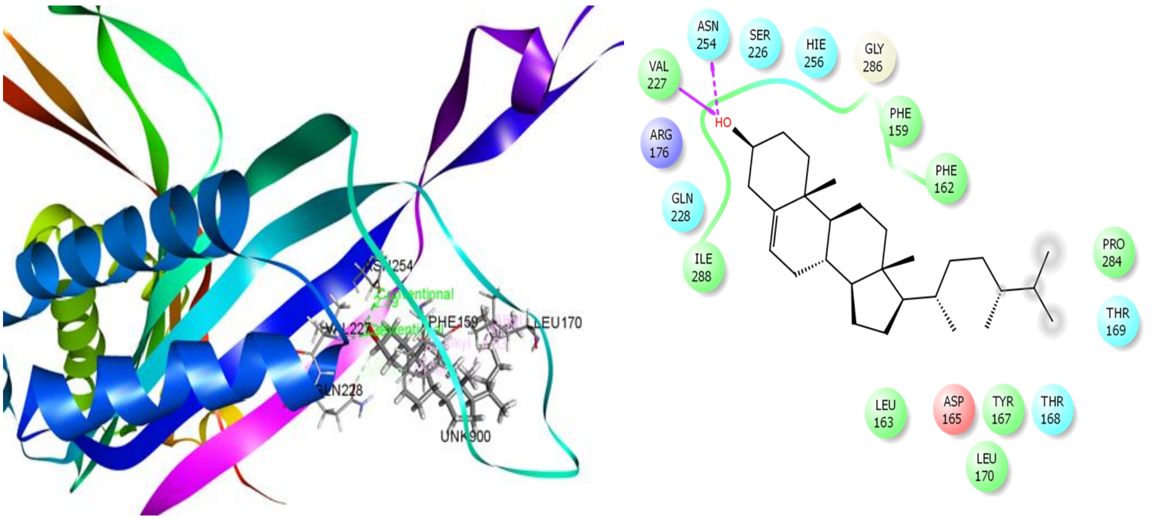

Figure S28. Best ranked poses and 2D interactions of campesterol with *xanthine oxidoreductase* (pdb: 1R4U) for antioxidant activity

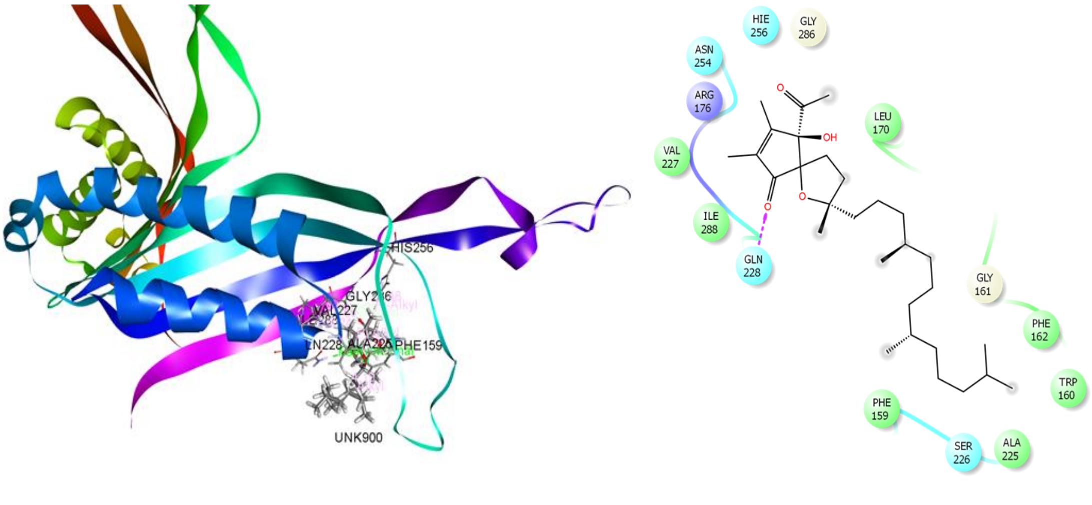

Figure S29. Best ranked poses and 2D interactions of alpha.-Tocospiro A with *xanthine oxidoreductase* (pdb: 1R4U) for antioxidant activity

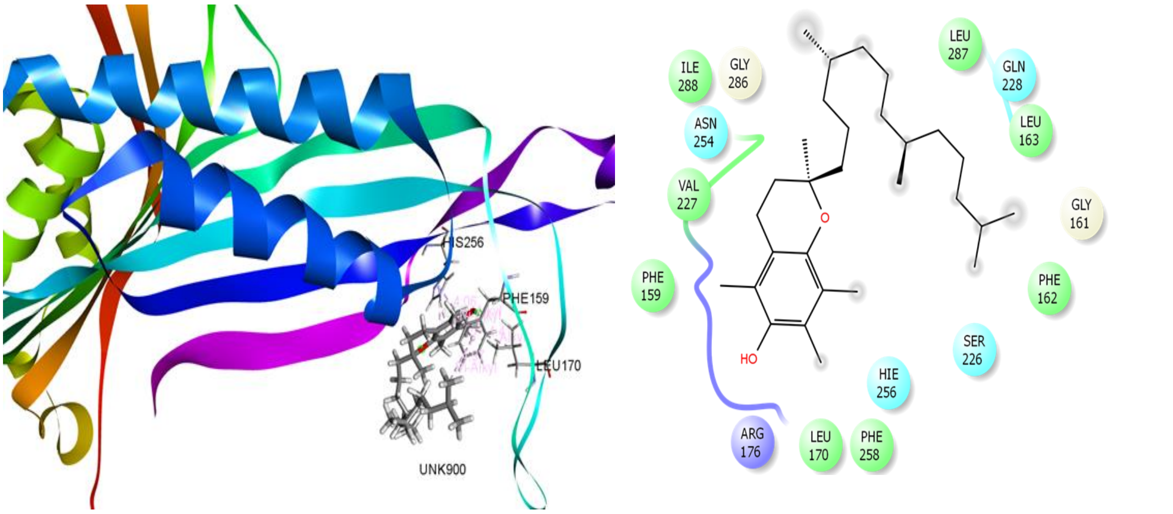

Figure S30. Best ranked poses and 2D interactions of vitamin E with *xanthine oxidoreductase* (pdb: 1R4U) for antioxidant activity

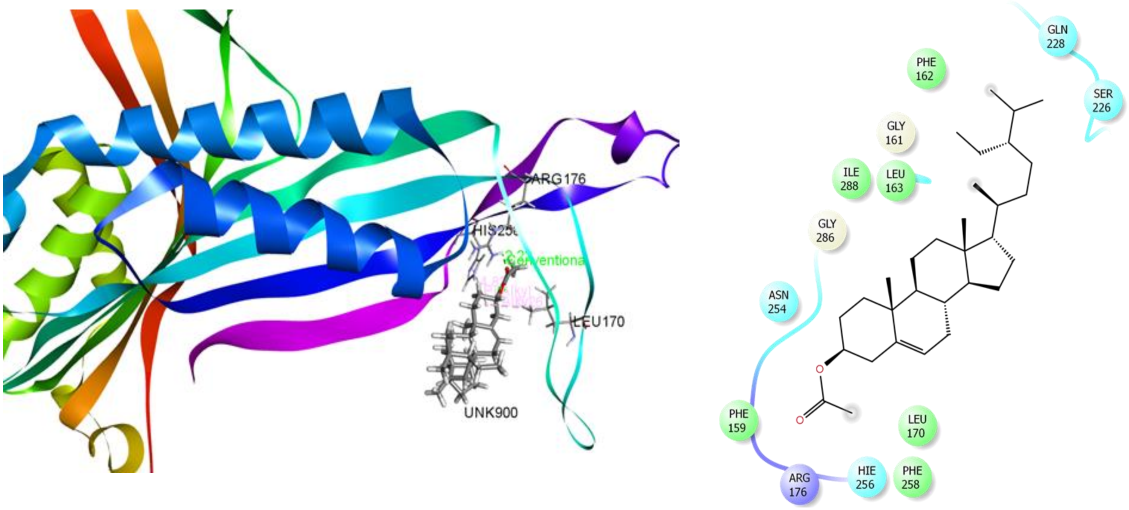

Figure S31. Best ranked poses and 2D interactions of beta.-Sitosterol acetate with *xanthine oxidoreductase* (pdb: 1R4U) for antioxidant activity

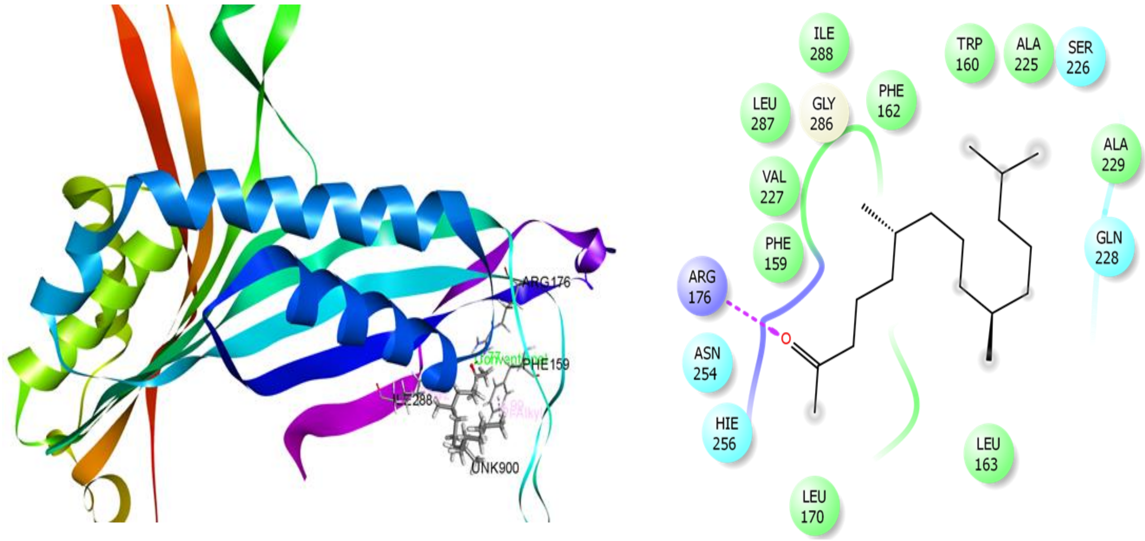

Figure S32. Best ranked poses and 2D interactions of 2-Pentadecanone, 6,10,14-trimethyl with *xanthine oxidoreductase* (pdb: 1R4U) for antioxidant activity

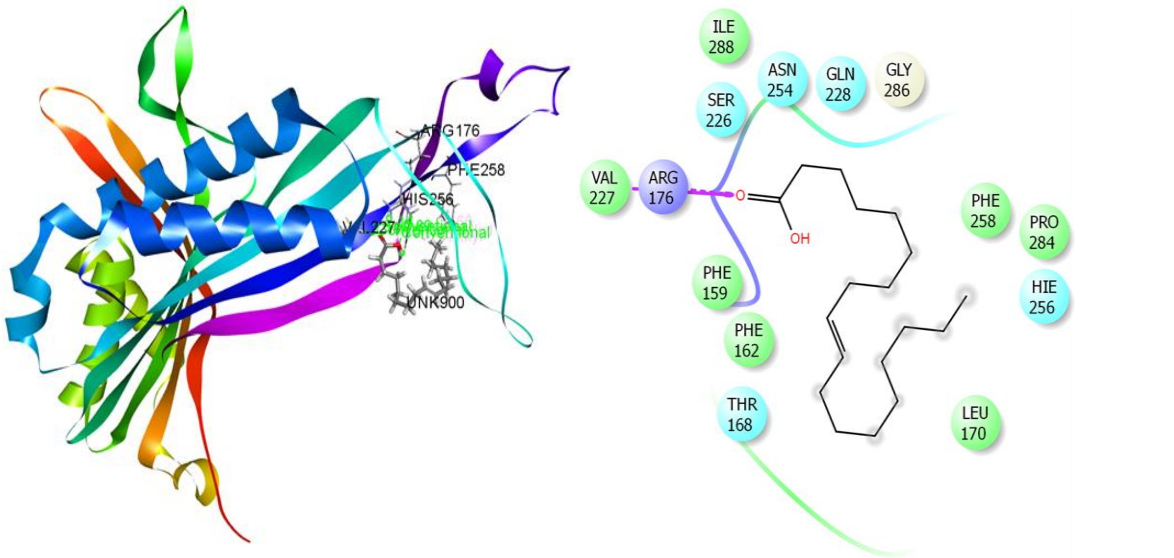

Figure S33. Best ranked poses and 2D interactions of Elaidic acid with *xanthine oxidoreductase* (pdb: 1R4U) for antioxidant activity

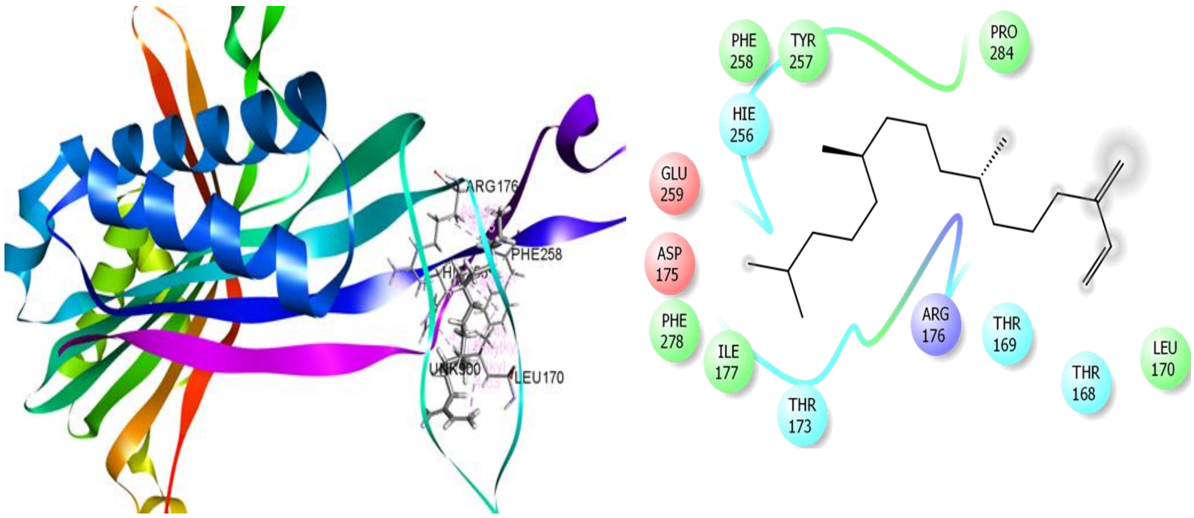

Figure S34. Best ranked poses and 2D interactions of Neophytadiene with *xanthine oxidoreductase* (pdb: 1R4U) for antioxidant activity

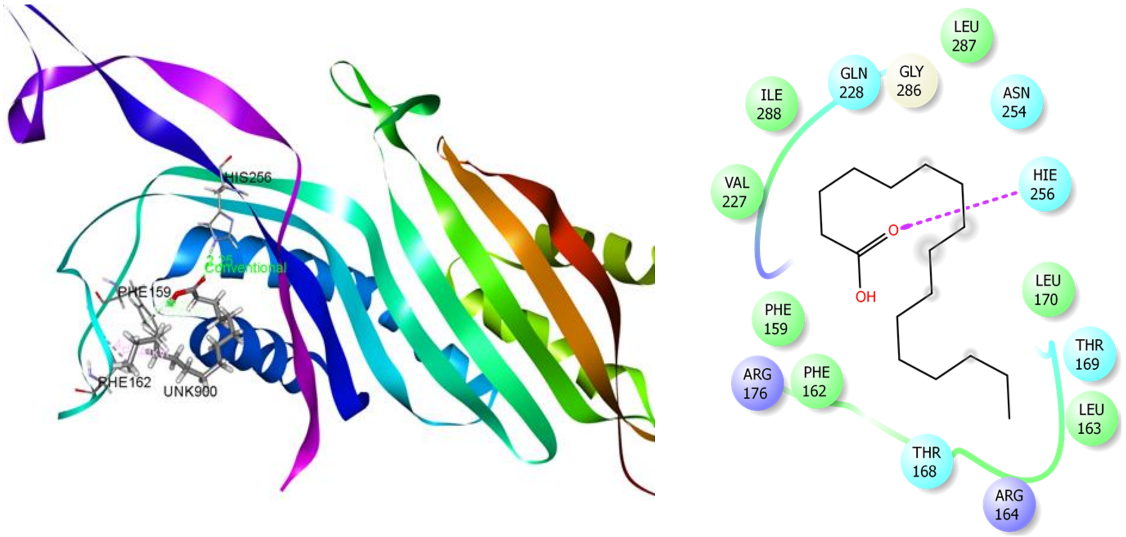

Figure S35. Best ranked poses and 2D interactions of n-Hexadecanoic acid with *xanthine oxidoreductase* (pdb: 1R4U) for antioxidant activity

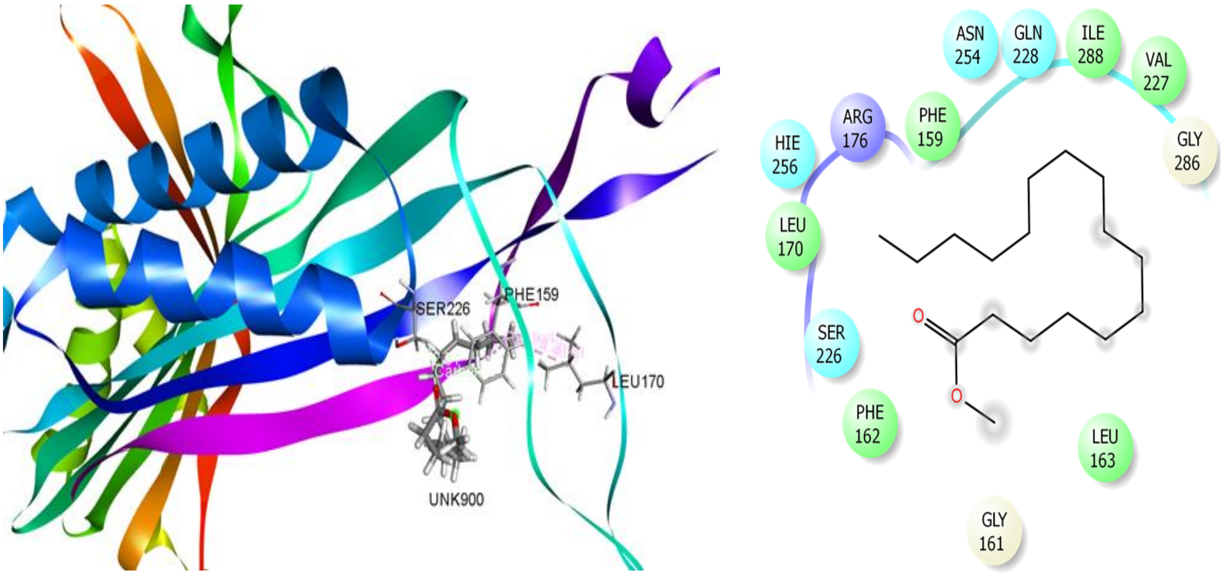

Figure S36. Best ranked poses and 2D interactions of Hexadecanoic acid, methyl ester with *xanthine oxidoreductase* (pdb: 1R4U) for antioxidant activity
